# Fluorescence Profiling of Urine in Non-Invasive Diagnostics of Obstructive and Reflux Uropathies in Children

**DOI:** 10.3390/medsci14060675

**Published:** 2026-10-15

**Authors:** Anastasiya A. Luenkova, Galina I. Kuzovleva, Marina A. Korshunova, Darya A. Perfilyeva, Ramil M. Akhmetov, Elena Yu. Danilova, Dmitrii A. Morozov, Mikhail K. Beklemishev, Olga L. Morozova

**Affiliations:** 1 Department of Chemistry, Lomonosov Moscow State University, Leninskie Gory 1–3, Moscow 119991, Russia; sovannarizz@gmail.com (A.A.L.); marina_ko_anat@mail.ru (M.A.K.); perfilevadaria@yandex.ru (D.A.P.); ramil.akhmetov.2004@gmail.com (R.M.A.); 2 Veltischev Research and Clinical Institute for Pediatrics and Pediatric Surgery, Pirogov Russian National Research Medical University, 2, Taldomskaya Street, Moscow 125412, Russia; kuzovlevagi@zdrav.mos.ru (G.I.K.); damorozov@list.ru (D.A.M.); 3 Speransky Children’s Hospital No 9, 29, Shmitovsky Proezd, Moscow 123317, Russia; 4 Molecular Theranostics Institute, Biomedical Science and Technology Park, I.M. Sechenov First Moscow State Medical University, 8, bld. 2b, Trubetskaya Street, Moscow 119991, Russia; danelui@ya.ru; 5 Department of Pathophysiology and Clinical Pathophysiology, Institute of Human Biology and Pathology, Russian National Research Medical University, 1, Ostrovityanova Street, Moscow 117997, Russia

**Keywords:** obstructive uropathies, urine metabolomics, reaction-based optical fingerprinting, chemometrics, pediatric urology

## Abstract

Background: Rapid screening methods reflecting the metabolic profile of urine are promising for assessing the risk of decreased renal function in children with obstructive uropathies. The aim of this pilot study was to evaluate the applicability of a reaction-based fingerprinting strategy for this purpose, which has previously demonstrated its effectiveness in recognizing samples with similar composition. Methods: The study included 28 children with hydronephrosis (HN, 5 patients) and vesicoureteral reflux (VUR, 15 patients), as well as healthy children without urinary pathology who comprised the control group (8 patients). Using the kinetic variant of optical fingerprinting, six indicator reactions of different natures involving dyes were investigated, with absorbance and fluorescence of the reaction products monitored photographically throughout the reaction. Results: Supervised chemometric analysis demonstrated that standard clinical and laboratory indicators enabled discrimination of urine samples from children with HN and VUR from the control group; however, the recognition accuracy was only satisfactory, ranging from 79% to 86%. The introduction of urine samples into the indicator reactions enabled discrimination of both diagnoses (VUR and HN) from the control group and from each other, with recognition accuracy reaching 100%. Furthermore, no correlation was observed between the results obtained using kinetic-based methods and standard clinical and laboratory indicators, which may reflect different levels of the pathological process, namely functional and molecular. Conclusions: The reaction-based optical fingerprinting strategy can be considered a promising approach for in-depth non-invasive diagnostics in pediatric urology. The reported diagnostic performance is limited to the analyzed cohort and should not be extrapolated to general clinical use without independent validation in a larger, age-matched population. If validated, kinetic optical fingerprinting could serve as an adjunct to conventional imaging and clinical assessment to inform risk stratification and guide further investigations, rather than functioning as a stand-alone decision-maker.

## 1. Introduction

Congenital Anomalies of the Kidney and Urinary Tract (CAKUT) comprise a heterogeneous group of structural abnormalities resulting from defects in urinary system embryogenesis during the critical period of 3–10 weeks of gestation. This term encompasses a broad spectrum of malformations, among which obstructive uropathies, including hydronephrosis (HR), megaureter, and vesicoureteral reflux (VUR) [1,2], are of particular clinical significance.

The major complications of obstructive and reflux uropathies include recurrent urinary tract infections (UTIs), nephrosclerosis, arterial hypertension, and progression of CKD. UTIs occurring in the setting of impaired urodynamics play an important role in the development of cicatricial changes in the renal parenchyma [3]. According to current studies, up to 40–50% of cases of end-stage CKD in children requiring renal replacement therapy are associated with CAKUT syndrome [4].

Despite the widespread use of instrumental diagnostic methods, including ultrasound examination, voiding cystourethrography, and radionuclide studies, assessment of the functional state of the kidneys and differentiation of obstructive and reflux processes remain challenging.

In recent years, increasing attention has been directed toward non-invasive diagnostic approaches based on alterations in the metabolome and proteome. These methods enable the detection of early biochemical changes associated with inflammation, oxidative stress, and fibrosis, which is particularly relevant in pediatric practice, where the use of invasive diagnostic procedures should be minimized [5,6,7].

To address these diagnostic gaps, pattern recognition methodologies (or «fingerprinting») have emerged, shifting the focus from quantifying individual biomarkers to capturing holistic biochemical profiles. Multisensor systems like the «electronic nose» (e-nose) can rapidly identify volatile organic compound (VOC) profiles linked to bacterial UTIs and lipid peroxidation products [8,9]. Similarly, the «electronic tongue» (e-tongue) has demonstrated utility in monitoring renal concentrating capacity, detecting microalbuminuria, and differentiating infectious from sterile inflammation [10,11]. Complementing these electrochemical approaches, fluorescence spectroscopy generates metabolic fingerprints by recording changes in endogenous fluorophores (e.g., tryptophan, NADH, porphyrins) triggered by hypoxia and tubulointerstitial fibrosis. The diagnostic yield can be further expanded by introducing exogenous fluorophores to map broader chemical interactions [12,13].

However, static profiling methods often face limitations in capturing the complex chemical composition of real-world biological samples. The specific novelty of the proposed method lies in transitioning to a kinetic optical fingerprinting approach based on conducting indicator reactions in the presence of the sample. Rather than measuring static intrinsic signals, this method records time-dependent changes in fluorescence and light absorption during a controlled chemical reaction, such as dye oxidation. Originally proven effective in differentiating complex biological matrices like sera of cancer-inoculated mice [14], this kinetic variant amplifies subtle biochemical variations that static methods may overlook. Applying this approach to urine samples offers a highly sensitive, entirely non-invasive diagnostic tool that could significantly decrease the need for ionizing radiation and painful procedures like bladder catheterization or venous access in children.

Currently, data on optical profiling in pediatric CAKUT remain limited, and standardized diagnostic criteria are lacking. We hypothesize that the distinct pathogenetic mechanisms of obstructive uropathies (driven by ischemia and fibrosis) and reflux uropathies (driven by chronic, low-intensity inflammation) induce specific metabolic alterations in urine that can be uniquely resolved via kinetic optical fingerprinting. Accordingly, the aim of this study was to evaluate the diagnostic capability of this novel reaction-based recognition approach for differentiating obstructive and reflux uropathies in children and assessing their risk of renal parenchymal damage.

## 2. Materials and Methods

### 2.1. Samples

The present prospective, single-center pilot study on urine profiling (approved by the Local Ethics Committee of Pirogov Russian National Research Medical University. No. 259 of 16 March 2026) involved 28 children aged 0.5–7 years (control group—up to 12 years), undergoing examination and treatment in a specialized hospital. In all groups, male patients predominated. Patients were divided into three groups depending on the clinical diagnosis. The control group consisted of 8 children without urinary pathology (healthy controls), admitted for planned treatment for conditions such as phimosis, hydrocele of the testis, inguinal hernias, and who had normal urine test results, including the absence of leukocyturia and bacteriuria. The second group included 15 patients with a confirmed diagnosis of vesicoureteral reflux (VUR). The third group consisted of 5 patients with hydronephrosis. Patient recruitment took place between 18 March 2026, and 30 June 2026; patients were enrolled consecutively. Inclusion criteria: age 0 to 12 years; confirmed diagnosis of obstructive uropathy (congenital hydronephrosis, vesicoureteral reflux); indication for surgical treatment; clinical and laboratory remission of urinary tract infection; signed informed consent from parents or legal representatives. Exclusion criteria: secondary megaureter or vesicoureteral reflux due to neurogenic bladder or posterior urethral valves; prior reconstructive surgery on the ureteropelvic junction or ureter; severe systemic diseases capable of affecting the urinary metabolomic profile; use of medications; refusal of parents or guardians to participate in the study. No eligible participants or samples were excluded from the analysis.

The patients were diagnosed with ureteropelvic junction obstruction (congenital hydronephrosis) and vesicoureteral reflux (Table 1). They underwent a standard clinical and laboratory examination, including history taking, physical examination, and standard laboratory methods. A complete blood count with determination of leukocyte count and erythrocyte sedimentation rate (ESR), a biochemical blood test with assessment of creatinine and urea concentrations, as well as determination of C-reactive protein level, were performed. Renal function was assessed by the estimated glomerular filtration rate (eGFR). A urinalysis with assessment of proteinuria and leukocyturia was also performed. Instrumental examination included renal ultrasonography with Doppler imaging, voiding cystourethrography (VCUG), excretory urography or contrast-enhanced computed tomography (CT) of the kidneys, magnetic resonance imaging (MRI), and static renal scintigraphy (when clinically indicated). Congenital hydronephrosis (ureteropelvic junction obstruction) was diagnosed when ultrasonography demonstrated pyelocaliectasis and renal parenchymal thinning and delayed urograms showed delayed clearance of contrast medium from the renal collecting system. If VCUG demonstrated reflux of contrast medium into the ureter and renal collecting system, the child was diagnosed with vesicoureteral reflux; the grade was determined according to the international classification of vesicoureteral reflux. Children receiving medication were not included in the study.

Biomaterial was collected in the morning after overnight fasting one day after admission, during which patients received a standard age-appropriate diet. A morning midstream urine sample collected in a sterile container was used. For urine collection in children under 1.5 years of age, adhesive urine collection bags attached to the perineal skin were used [15]. Samples were allowed to settle for 2 h at room temperature to remove cellular elements, after which the supernatant was collected with a sterile syringe, transferred to Eppendorf tubes, labeled, and stored in a freezer at −20 °C until analysis. The researchers were not formally blinded; however, the analysis process was identical for all wells on the plate. For the same indicator reaction, all samples were measured simultaneously (on the same plate), thereby minimizing the influence of factors such as the analytical batch or the date of measurement.

### 2.2. Methodology, Reagents and Instruments

**Figure 1:**
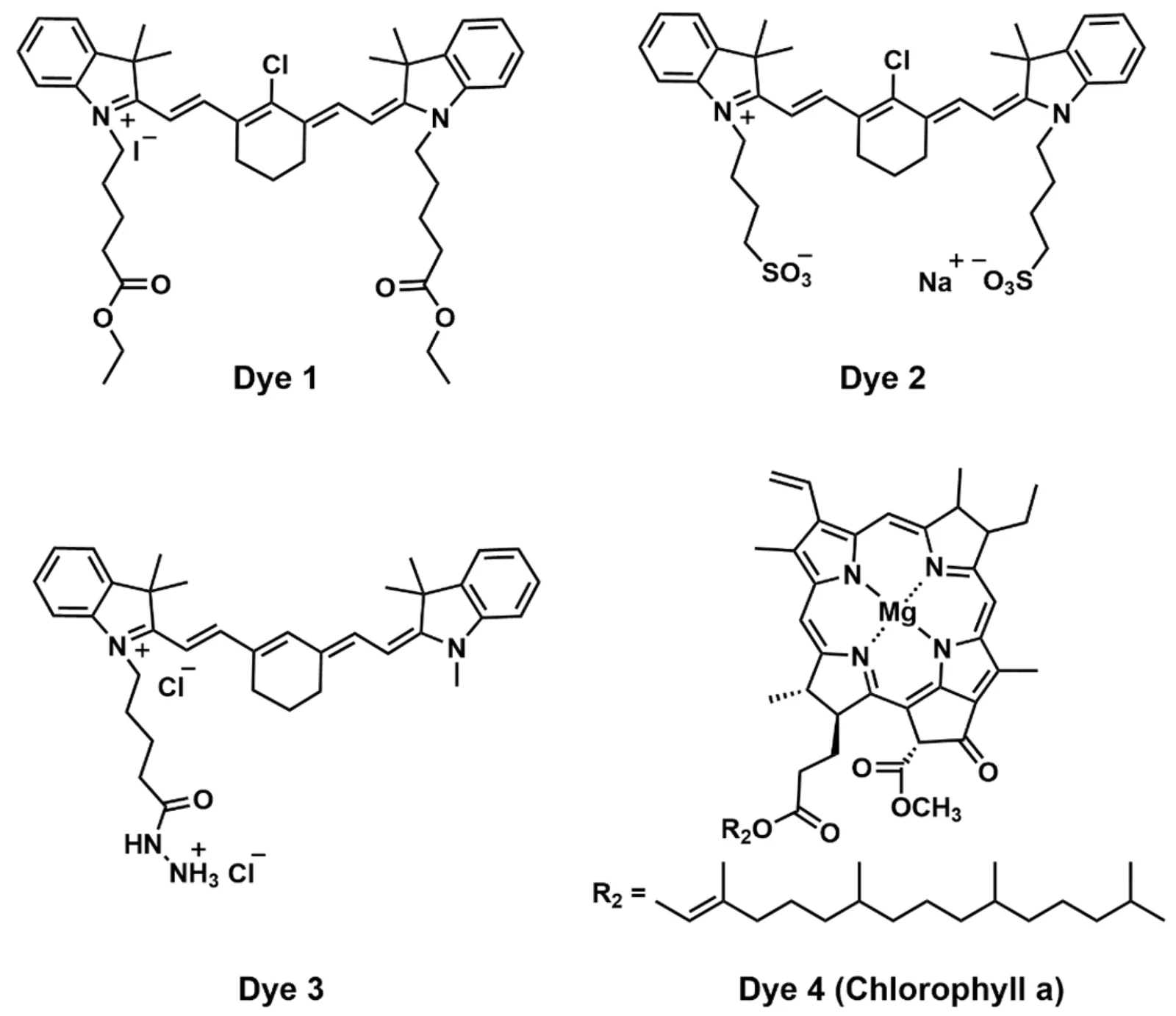
Structures of the dyes.

When studying urine by the kinetic “fingerprint” method, samples were thawed by immersing the lower half of the tubes in water at room temperature. After thawing, 10 μL of the sample was introduced into a 96-well plate using a dispenser, into which the components of the indicator systems were also placed (conditions are given in Table 2). Each sample was tested in six replicates (in reaction A, in four replicates). Only one indicator reaction was carried out in one plate at a time. The moment of oxidant addition was taken as the reaction start time. In reactions A, B, C, D, and F, carbocyanine dyes were used (Figure 1). Dyes 1 and 2 were synthesized by the authors according to [16] and [17], respectively. Dye 3 was purchased from Lumiprobe (Lumiprobe, Hunt Valley, MD, USA). Gold nanoclusters stabilized with adenosine monophosphate [18] were used as catalysts for the oxidation of carbocyanine 1 by hydrogen peroxide (reaction F). In reaction E, chlorophyll isolated by the authors from spinach according to [19] was oxidized by hydrogen peroxide (Komponent-Reaktiv LLC, Moscow, Russia). Riboflavin (Sigma-Aldrich, St. Louis, MO, USA) was used as a photosensitizer in the oxidation of dye 1 by peroxide (Figure 1, reaction B).

Sigma-Aldrich reagents were used without further purification. Acetate buffer solutions were 0.1 M in acetate, and phosphate buffer solutions were 0.067 M in phosphate. Deionized water purified with an Ulupure system (Chengdu Ultrapure Technology, Chengdu, China), conductivity 18.2 MΩ·cm, was used to prepare solutions. Carbocyanine dyes were dissolved in 95% ethanol (Bryntsalov-A, Moscow, Russia) at a concentration of 1 mg/mL and stored at 4 °C; diluted solutions were prepared on the day of the experiment by diluting the stock solutions with water.

**Figure 2:**
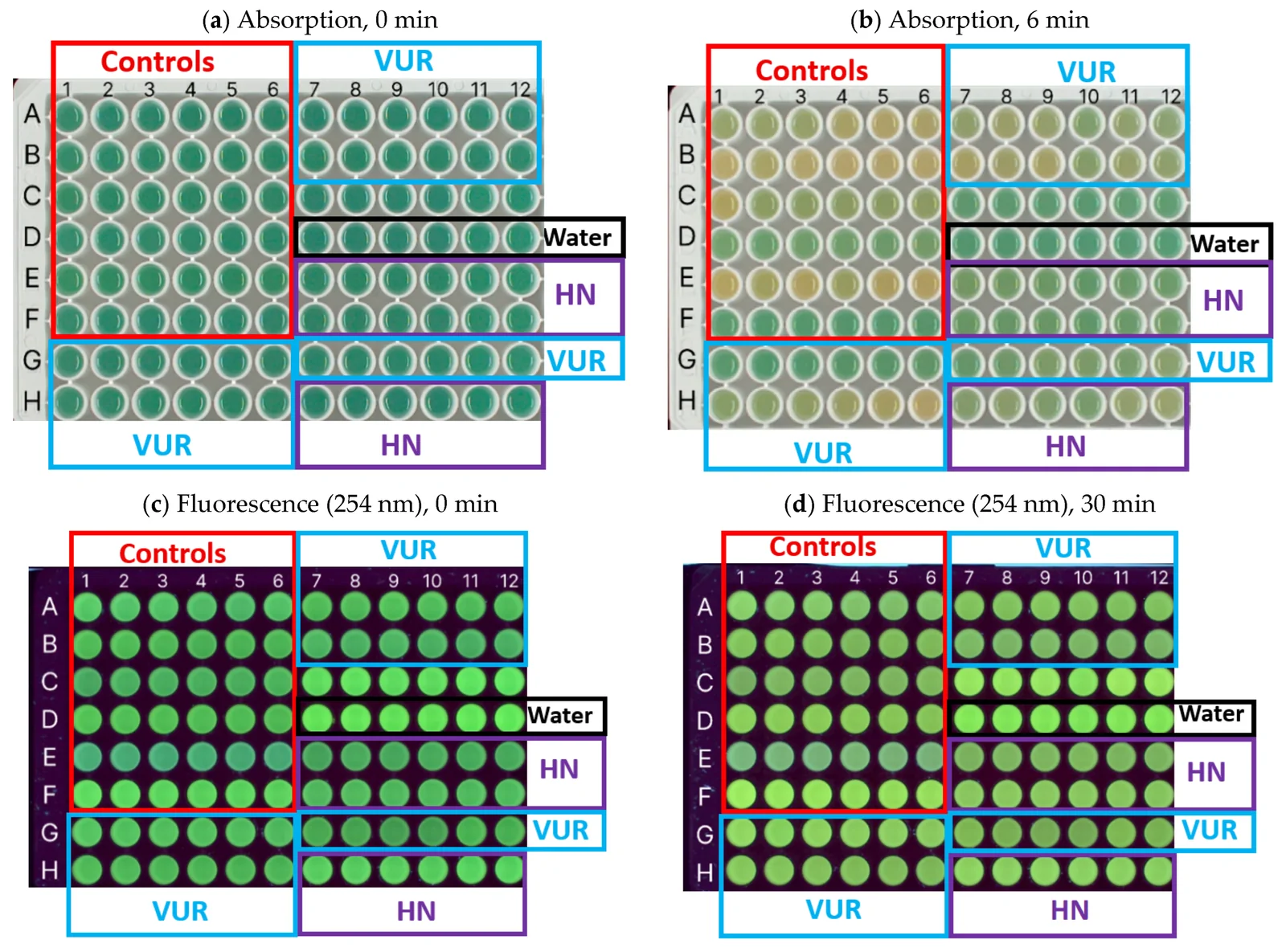
Photographs of 96-well plates with reaction mixtures (indicator reaction B) at different time points: (**a**,**b**) visible images, (**c**,**d**) fluorescence excited at 254 nm. Wells containing urine samples from children with hydronephrosis (HN), vesicoureteral reflux (VUR), and the control group, as well as water, are indicated next to the images. Note. The unlabeled sample C7…C12 was not processed.

**Figure 3:**
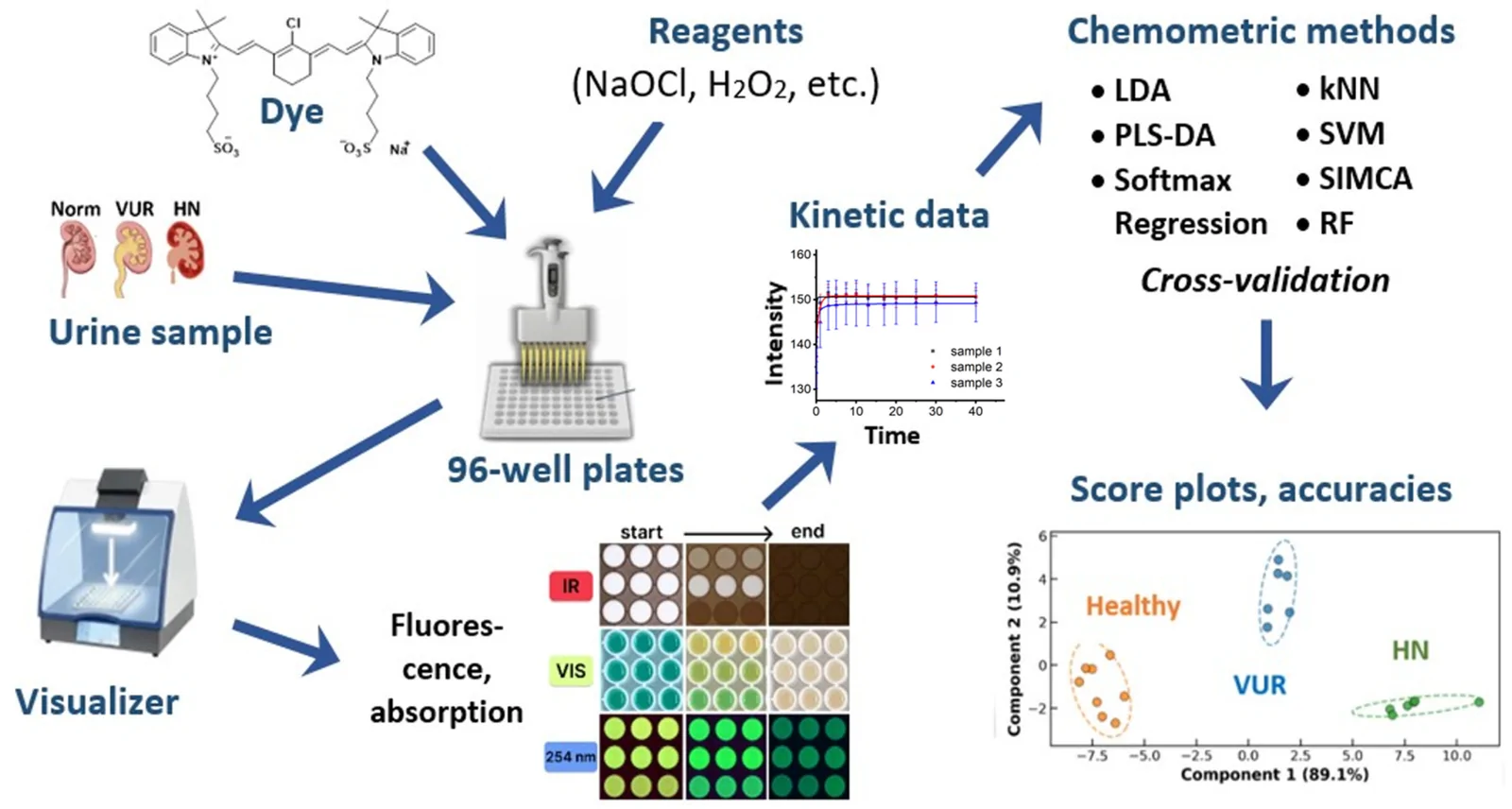
General outline of recognizing uropathies using kinetic optical fingerprinting.

After the start of the indicator reaction, the plate was photographed in different spectral ranges (1–4), depending on the reaction used (Table 2): (1) absorption/reflection of visible light; (2) fluorescence in the visible region excited at 254 nm; (3) the same, with excitation at 366 nm; (4) fluorescence in the near-IR region excited at 660 nm. Photographic images of the plate in ranges (1–3) were obtained using a Visualizer 2 (Camag, Muttenz, Switzerland), and in range (4) using a homemade visualizer in which red LEDs served as the light source, and photographs were taken with a Nikon D50 (Nikon, Tokyo, Japan) or Canon EOS 350D camera (Canon, Kunisaki, Japan.) equipped with a 720 nm long-pass filter [20]. As the indicator reaction proceeded, photographs of the plate in each spectral range were obtained over 0.5–1.5 h. Examples of photographs of the plate with reaction mixtures are shown in Figure 2, and the general scheme of the experiment is given in Figure 3.

### 2.3. Data Processing

Photographs were digitized using the cloud-based software Chrometrica.ru developed by the authors [21]. The photographs were either subjected to RGB splitting, yielding three intensity channels, red, green, and blue (R, G, B), for each photo, or the overall image intensities were used (as specified in the text). The resulting intensity values for each indicator reaction were compiled into data tables in which the rows corresponded to samples (observations) and the columns corresponded to photographs taken at different time points in different spectral channels (depending on the nature of the indicator reaction, from 7 to 23 photographs were obtained over the entire reaction).

The data were processed using chemometric methods: principal component analysis (as an unsupervised method of data compression) and supervised methods with the following default parameter values: linear discriminant analysis (LDA) based on singular value decomposition with the number of discriminant components set to the minimum of the number of classes minus one and the number of features; softmax regression (SR) with L2 regularization, an inverse regularization strength equal to 1, and a maximum of 1000 iterations; k-nearest neighbors (kNN) with 5 neighbors, the Minkowski distance metric, and the Euclidean power parameter set to 2; a support vector machine classifier (SVM) with the inverse regularization strength fixed at 1; a random forest (RF) comprising 100 decision trees; partial least squares discriminant analysis (PLS-DA) in its hard classification version with varied number of latent variables to be optimized (2–9) and an error rate of 0.05; and soft independent modelling of class analogy (SIMCA) with 7 principal components and the same error rate. For logistic regression, kNN, PLS-DA, and SIMCA, the corresponding feature matrices were centered and scaled to unit variance prior to modelling.

To build chemometric models, the intensity values of replicate measurements were averaged for each urine sample (typically 6 replicates were used; accordingly, the number of rows in the data table was reduced by a factor of 6). Although repeated measurements can improve measurement precision, they do not increase the effective clinical sample size. Feature selection (including spectral range, number of images, and averaging) was performed predominantly manually using the entire dataset. In addition, an automatic feature selection function was developed and applied for comparison; the corresponding results are presented in Supplementary Table S5.

As first stage of data processing, the quality of classification was assessed using Chrometrica software by leave-one-out cross-validation, sequentially removing each sample from the model and calculating the overall classification accuracy (ACC) and, in the case of LDA, the area under the ROC curve (AUC). The averaged ACC or AUC values over all cross-validation folds are reported below as the number of correctly classified samples divided by the total number of samples: ACC = (TP + TN)/(TP + TN + FP + FN), where TP is the number of true positives (sick), TN—true negatives (controls), FP—false positives, FN—false negatives. ACC values in the range of 90–99% were considered “good,” 80–89% “satisfactory,” and below 80% “unsatisfactory” [22].

In most cases, sample discrimination was performed for two classes (e.g., all samples with uropathies vs. controls, or only samples with hydronephrosis vs. controls, etc.). Sensitivity (SENS) and specificity (SPEC) metrics were calculated using the standard formulas SENS = TP/(TP + FN), SPEC = TN/(TN + FP).

At the second stage of data processing, we manually selected the spectral range, RGB averaging and the number of images. The initial spectral range was predetermined by the photophysical properties of each dye, most of which were NIR-emitting carbocyanines, while riboflavin also fluoresced in the visible region; visible absorption images were acquired for nearly all experiments. After data collection, we systematically compared all combinations of spectral range and processing type (RGB-splitting vs. total intensity) for each reaction and classification task, selecting the combination with the highest accuracy and, in case of ties, the one requiring fewer features. For that optimal combination, we then varied the number of photographs, reducing it only when accuracy improved. Model parameters were kept at default values, except for PLS-DA, where the number of latent variables was optimized between 2 and 9. This selection was performed on the full dataset, which may introduce optimistic bias, and we are explicitly stating this as a limitation.

To quantify the uncertainty of the classification performance obtained for each task, 95% confidence intervals (CIs) were calculated from the pooled out-of-fold predictions of leave-one-out cross-validation (LOOCV). For accuracy, sensitivity and specificity, the Clopper–Pearson exact method was used, which is based on the binomial distribution and guarantees coverage at or above the nominal level regardless of sample size; this is particularly important for the small pilot cohort and for values approaching 100%. The CI of balanced accuracy (the mean of sensitivity and specificity) was obtained by the MOVER method from the Clopper–Pearson intervals of the two class-wise recalls. For cross-validated AUC, to which the Clopper–Pearson method is not applicable, the score-type interval of Newcombe based on the Hanley–McNeil variance was used; unlike Wald-type intervals, it does not degenerate when the AUC equals 1. To assess the risk of overfitting and the statistical significance of the PLS-DA and LDA models, a permutation test (*y*-scrambling) was applied [23]. The test procedure consisted in randomly permuting (shuffling) the class label vector (Y) while keeping the feature matrix unchanged. For each of 200 permutations, the model (PLS-DA or LDA) was retrained using the same cross-validation scheme as for the original model, and the corresponding quality metrics were computed: for PLS-DA, the coefficient of determination R^2^ (on calibration data) and the predictive ability coefficient Q^2^ (from cross-validation results); for LDA, classification accuracy and/or AUC, also calculated on the calibration and cross-validation sets. The values obtained when training the model on unshuffled (original) data were referred to as “original.” For each permutation, the Pearson correlation coefficient between the permuted and original Y vector was additionally calculated. Based on the obtained points, a plot of model quality metrics versus the correlation coefficient with the original Y vector (permutation plot) was constructed; a regression line was fitted through the point cloud by the least squares method and extrapolated to the intersection with the ordinate axis (at a correlation score of 0), yielding the intercept value. For PLS-DA, a model was considered statistically significant and not overfitted if: (i) the original R^2^ and Q^2^ values exceeded 0.6; (ii) the Q^2^ intercept was negative. For LDA models, a permutation plot with regression lines and intercepts was constructed for AUC metric, and the original values were compared with the distribution of values obtained from permutations; the magnitude of the intercepts were also examined, with values close to 0.5 indicating the absence of overfitting, whereas values approaching 1 suggested overfitting. In addition, to assess the degree of overfitting of the LDA and PLS-DA models, both standard permutation *p*-values and cross-validated *p*-values were calculated. The cross-validated *p*-value was defined as the fraction of permutations yielding a metric equal to or more extreme than the original quality metric obtained from cross-validation (CV AUC for LDA and Q^2^ for PLS-DA). Given the small sample size, standard permutation *p*-values almost invariably indicated statistical insignificance due to limited statistical power. Therefore, greater emphasis was placed on the cross-validated *p*-values for assessing the risk of overfitting. A cross-validated *p*-value below 0.05 was considered indicative of the absence of overfitting, although it should not be regarded as definitive proof. All calculations were performed using the chrometrica.ru website.

## 3. Results

### 3.1. Use of Standard Clinical and Laboratory Parameters for the Recognition of Uropathies

To compare patient groups based on standard clinical and laboratory parameters using chemometric methods, data tables were compiled with samples represented by rows and variables by columns. The variables included age (years), sex (M—1, F—2), eGFR (mL/min/1.73 m^2^), and the following complete blood count parameters: leukocytes (×10^3^ µL^–1^), ESR (mm/h), urea (mmol/L), and creatinine (µmol/L) (Table 1). These parameters were selected because they were available for nearly all subjects. For the 9 missing values out of 189, the corresponding column mean was used. Proteinuria, leukocyturia, and elevated C-reactive protein were observed in only 1–3 patients and were therefore excluded from further analysis.

Principal component analysis of the initial data structure (Figure S1 in ESI) showed that the urine samples could not be reliably distinguished according to class (control, hydronephrosis, and VUR) based on standard clinical and laboratory parameters. Therefore, supervised chemometric methods were subsequently applied (Table 3). Discrimination of the samples into three classes according to diagnosis (control, hydronephrosis, and VUR) did not provide a satisfactory result, with an accuracy not exceeding 71%. Two-class discrimination performed better for samples from patients with VUR, which could be distinguished both from controls and from all other samples (control and hydronephrosis); however, the accuracy remained only satisfactory, not exceeding 86%. The two diagnoses, hydronephrosis and VUR, could be distinguished from each other with satisfactory accuracy (80%). In contrast, satisfactory discrimination could not be achieved when hydronephrosis samples were compared with either the control group or the combined [control + VUR] group, for which the accuracy did not exceed 73%. Thus, standard laboratory methods allowed discrimination only of VUR, and even in this case the accuracy was merely satisfactory.

### 3.2. Use of the Kinetic Optical Fingerprinting Method for the Recognition of Uropathies

#### 3.2.1. Indicator Systems

The described variant of the optical method for recognizing samples of similar composition is based on the influence of sample components on the shape of the kinetic curve of an indicator reaction [14,24,25]. To encompass as broad a range of influencing compounds as possible, processes of different natures are tested as indicator reactions (Table 2). The sample (in this case, untreated patient urine) is mixed with the reaction components, and light absorption and fluorescence are measured in different spectral ranges by periodically photographing the contents of the wells of a 96-well plate. The intensity values obtained by digitizing the images are used as optical fingerprints of the samples, which can then be discriminated using chemometric methods. Such discrimination is based on the fact that samples differ in their metabolomic composition and that different compounds affect the course of indicator reactions in different ways. The mechanisms of such influence may include binding to the catalyst, scavenging of intermediate species (active centers) formed during radical chain reactions, and other processes.

Let us characterize the indicator reactions used in the study. Reactions A and C represent the oxidation of carbocyanine dyes 1 and 2 by hydrogen peroxide and hypochlorite, respectively, which have previously proven effective for the recognition of samples of various compositions [22]. Reaction B also involves the oxidation of dye 2; however, in this system, oxidation occurs through the photochemical generation of reactive oxygen species by photoexcited riboflavin. For this purpose, the system is irradiated with blue light (450 nm) during the reaction [26]. In reaction D, carbocyanine dye 3, bearing an attached hydrazide group, was used. This group interacts with *o*-phthalaldehyde (PDA) to form an azomethine [27]. In reaction E, chlorophyll isolated from spinach was used as the dye; this natural pigment had previously been used by us as an analytical reagent in paper [19]. Reaction F represents the oxidation of dye 1 by peroxide catalyzed by gold nanoclusters [18], which exhibit high reactivity toward organic compounds [28] and are capable of changing their catalytic activity under their influence.

#### 3.2.2. Selection of Data Processing Conditions

**Figure 4:**
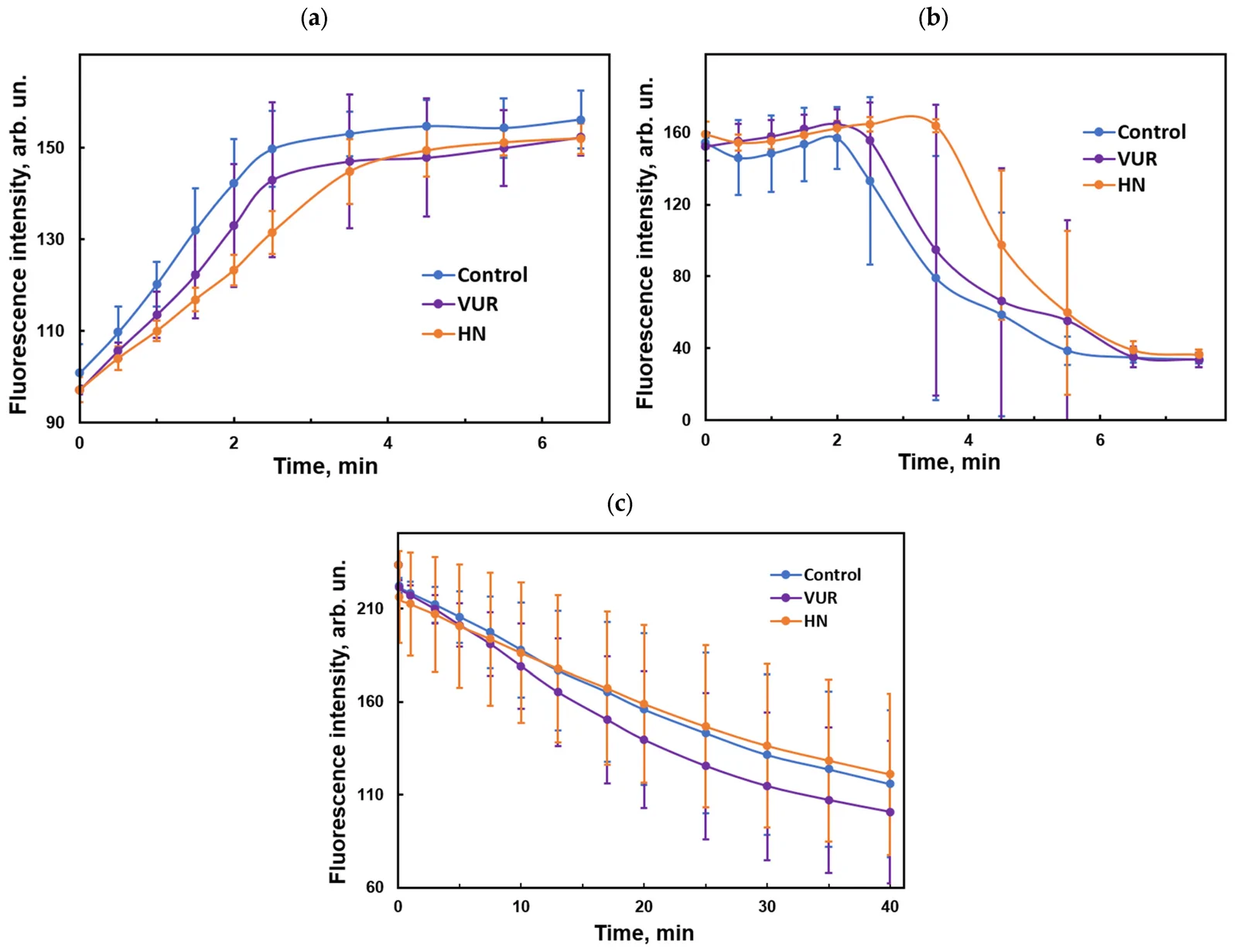
Kinetic curves: (**a**) indicator reaction B, visible fluorescence excited at 366 nm; (**b**) reaction B, fluorescence in the NIR region excited at 660 nm; (**c**) reaction C, fluorescence in the NIR region excited at 660 nm; VUR—vesicoureteral reflux, HN—hydronephrosis. Error bars correspond to a standard deviation.

To recognize urine samples from children with uropathies and control samples, indicator reactions A–F were carried out in the presence of the samples. The reaction mixtures in the plate were photographed in four spectral ranges: visible absorption and fluorescence excited at 254, 366, and 660 nm. Examples of the resulting plate images are presented in Figure 2, while examples of the results obtained by digitizing the photographs (kinetic reaction curves) in the presence of urine samples are shown in Figure 4.

**Figure 5:**
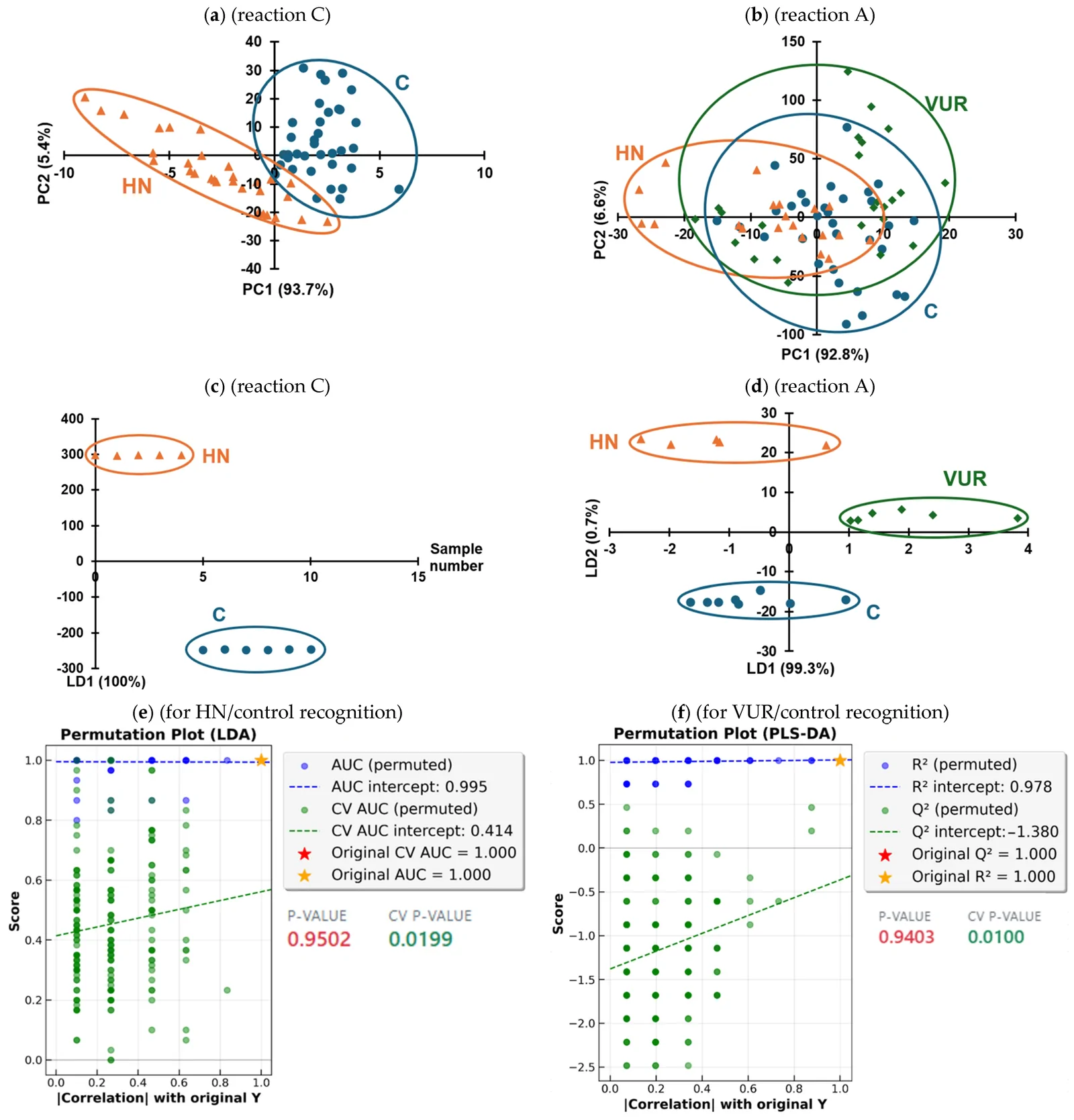
Score plots of principal component analysis for distinguishing samples with hydronephrosis from control samples by reaction C (**a**) and for separating three classes of samples: with hydronephrosis, with VUR, and control by reaction A using 12 photographs of the plate in the NIR range (**b**); the dependence of the first LDA factor on sample number corresponding to panel *a* (**c**); the LDA score plot corresponding to panel *b* (**d**); permutation test plots for checking the degree of model overfitting: (**e**) for distinguishing hydronephrosis and control samples using reaction C (accuracy 100% by the LDA method, AUC 1.00; 9 photographs of visible absorption of the plate without RGB-splitting were used); (**f**) for recognition of samples with VUR and control samples using reaction A (accuracy 100% by the PLS-DA method; 23 photographs of the plate in the NIR range were used). The red star is located exactly behind the yellow star because their values are identical for both subfigures (**e**) and (**f**).

The score plots of principal component analysis (an unsupervised method; Figure 5a,b) show that simple data compression is insufficient for recognizing uropathies and that supervised methods are therefore required. Further processing of the photographic data was performed using the 7 most common chemometric methods that have previously proven effective for recognizing samples of similar composition [29]. At the first stage, the data were processed automatically using the chrometrica.ru package (freeware) with the default method parameters specified in Section 2, using the spectral ranges and processing type indicated in Table 2 for each reaction. The results showed that hydronephrosis could be distinguished from control samples with an accuracy of 98–99% and from VUR with an accuracy of 82–84% (Table S1). However, the results of the automated processing cannot be considered final for the following reasons.

First, recognition accuracy can be improved by manually varying the following parameters: (a) the spectral ranges subjected to processing, (b) the type of intensity values (overall intensity or RGB splitting of the image, hereinafter referred to as the “processing type”), and (c) the number of photographs processed for each reaction by excluding some of them. Second, it is necessary, especially when high accuracy values are obtained, to check the chemometric model for overfitting, which is frequently observed when the number of samples used is limited [30].

In view of the above, the conditions (a–c): spectral range, processing type, and number of photographs included in the data processing—were selected manually using the full dataset, with the criterion being the maximum recognition accuracy while ensuring that no signs of overfitting were observed. The results showed that there is no universal spectral range (a) or processing type (b); rather, these parameters must be selected separately for each classification task. (Thus, the optimal processing conditions differ, for example, when distinguishing VUR from control samples and hydronephrosis from non-hydronephrosis samples.) An example of the effect of the selected spectral range and processing type on the results is given in Table S2. It can be seen that high recognition accuracy (up to 100%) for VUR and hydronephrosis is generally achieved using LDA and PLS-DA methods. However, in the case of VUR recognition, this requires processing photographs of visible absorption, whereas for hydronephrosis recognition, photographs of fluorescence in the IR range are required. Thus, the maximum accuracy for different recognition tasks is achieved using different spectral ranges and processing types.

The number of photographs included in the processing also affects the recognition results. During the course of the indicator reaction, 7 to 23 photographs were obtained, depending on the nature of the reaction. However, many of the images contain duplicate information and essentially introduce noise; therefore, their removal can improve recognition accuracy. For example, using every third image increased the recognition accuracy of VUR using reaction A by the LDA method from 67% (processing of all photographs) to 100% (processing of every third photograph, Table 4). At the same time, for the PLS-DA method, 100% accuracy was achieved both when all photographs were processed and when every second or third photograph was used, after which the accuracy decreased. The effect of the number of processed images on recognition accuracy for reaction C is further illustrated in Table S3, which shows that in some cases the accuracy remains virtually unchanged regardless of the number of photographs processed.

It should also be taken into account that changing the number of processed photographs can alter the results of permutation tests even when the accuracy remains unchanged. In the case of reaction A, reducing the number of processed photographs from 23 to 8 did not compromise the significance of the permutation test: the CV *p*-value remained 0.01 in all cases (Table S8). However, the R^2^ intercept decreased from 0.98 (23 images) to 0.25 (8 images), while the Q^2^ intercept remained negative (from −1.38 to −1.45). Thus, while the number of images affects the calibration intercepts, the cross-validated significance is stable for this task.

We also compared manual and automatic feature selection for HN vs. control discrimination using reaction C (Table S5). In the manual approach, every third photograph of the visible range was removed, leaving nine images; this yielded a CV *p*-value of 0.02 and a CV AUC intercept of 0.41. For automatic selection, we used the Chrometrica software, which ranks features by the ratio of between-class to within-class variance and retains the highest-ranked ones. With nine automatically selected features, the CV *p*-value was 0.04 and the CV AUC intercept was 0.41, comparable to manual selection. Reducing the automatically selected feature set to three yielded a lower CV *p*-value of 0.005 and a CV AUC intercept of 0.33. Thus, automatic feature selection did not uniformly outperform manual selection, but at a reduced feature count (three images) it produced a lower CV *p*-value and a lower intercept. Given the pilot nature of this study and the very small sample size, the manual procedure was retained as the primary approach, and the automatic results are presented only as a sensitivity check.

In addition to the above-described factors (a–c), the recognition results may be affected by whether replicate measurements of samples are averaged. The results showed that averaging can either improve or reduce recognition accuracy under otherwise identical processing conditions. Only in five recognition tasks (for reactions D and E) was the maximum accuracy achieved without averaging; however, it did not exceed 80% and was therefore considered unsatisfactory according to our criteria. Consequently, we excluded processing without averaging and present in the text only the values obtained by averaging the replicate measurements for each sample.

The selected conditions for processing the urine samples are summarized in Table 5, while the corresponding accuracy values for the recognition of uropathies under these conditions, together with the areas under the ROC curve obtained during cross-validation (CV AUC), are presented in Table 6. The highest accuracy values after optimization were obtained using the LDA and PLS-DA methods; the other methods yielded lower values. Therefore, Table 6 presents only the results obtained using LDA or PLS-DA. AUC values are provided for those systems in which the LDA method yielded higher accuracy, since overfitting of the LDA model was assessed using permutation tests with calculation of the AUC.

The machine learning models (LDA, PLS-DA, etc.) trained on the optical data in kinetic fingerprinting (Section 3.2) did not receive age or sex as input features. The feature matrices for these models consisted solely of digitized fluorescence and absorption intensities obtained from the 96-well plate photographs over time (Section 2.3). Therefore, the high discrimination accuracy (up to 100%, Table 6) is derived exclusively from the metabolic/chemical composition of the urine samples, not from explicit demographic labels. However, though the optical models did not “know” the patients’ age or sex, we acknowledge that age and sex are biological variables that significantly influence urine metabolome composition (e.g., due to hormonal differences, feeding patterns in younger children, and age-related metabolic maturation). Because our control group was older (up to 12 years) and single-sex, while the VUR and HN groups were significantly younger (0.5–7 years) and mixed-sex, there is a risk that the optical models are partially detecting age- or sex-specific metabolic patterns rather than purely disease-specific ones. This is a valid limitation of our small pilot dataset.

#### 3.2.3. Results of Recognition of Uropathies by the Kinetic Optical Fingerprinting Method

The obtained accuracy values are quite high. Thus, each of the two diagnoses (VUR and hydronephrosis), as well as the two diagnoses considered together, can be distinguished from the control group with an accuracy of 100%. Samples with hydronephrosis can likewise be distinguished from all other samples with 100% accuracy. In contrast, samples with VUR can be distinguished from all other samples only with “good” accuracy (93%), and only using reaction C. This can be explained by the fact that distinguishing the two uropathies from each other proved to be a more difficult task. Whereas samples with hydronephrosis could be separated from the control group using 4 indicator reactions, 100% accuracy in distinguishing the two uropathies from each other was achieved with only two indicator reactions. The attempt to recognize all three classes simultaneously (VUR, hydronephrosis, and the control group) was also successful, with an accuracy of 100%; however, this result was again achieved using only two indicator reactions.

Methods with diagnostic value are usually characterized by sensitivity, as a measure of the absence of false-negative results, and specificity, as a measure of the absence of false-positive results. However, when the accuracy is 100% (Table 6), both characteristics are, naturally, also equal to 100%, making the sensitivity and specificity values uninformative. In the case where the accuracy was 93% (recognition of VUR and all other samples), the sensitivity and specificity were 100% and 91%, respectively. The exact numbers of correctly and incorrectly classified cases and the 95% confidence intervals for the main performance metrics (accuracy, balanced accuracy, AUC, sensitivity, and specificity) are presented in Table S4. For binomial proportions (accuracy, sensitivity, and specificity), the Clopper–Pearson exact method was used, as it guarantees coverage at or above the nominal level even for small samples and extreme proportions. For the cross-validated AUC, we applied the score-type interval of Newcombe, which is based on the Hanley–McNeil variance and provides reliable intervals for small datasets, unlike Wald-type intervals that degenerate when AUC equals 1. The considerable width of the confidence intervals is attributable to the small sample size in this pilot study and should not be interpreted as a flaw in the model performance.

It is important to note that the high accuracy values, especially 100%, as well as the area under the curve values equal to unity obtained using LDA and PLS-DA, could indicate overfitting of the models. Therefore, permutation tests were performed for all recognition tasks: for PLS-DA, based on the Q^2^ criterion, and for LDA, based on the area under the ROC curve (AUC). Table 6 presents only the values that passed the overfitting check; the corresponding procedure for assessing model quality is described in Section 2.3. Examples of permutation plots are shown in Figure 5 (a more complete set of plots corresponding to the results in Table 6 is provided in Table S6). Thus, for the LDA method (Figure 5e), the AUC obtained by cross-validation was 1.0, whereas for fully permuted data (at *x* = 0), it was 0.41, which corresponds to statistical guessing. In some data permutations, the AUC still reached a value of 1.0, however, the fraction of such permutations, *p*, was small (for the total number of permutations of 200) indicating that the original model’s performance is statistically significant (*p* < 0.05). For the PLS-DA method (Figure 5f), the permuted R^2^ values remained high (intercept 0.978), whereas the permuted Q^2^ values decreased and were mostly negative (intercept −1.380). The original Q^2^ and R^2^ were both 1.000. Although the original model achieves perfect separation (R^2^ = Q^2^ = 1.000), such a result is itself a reason to suspect overfitting, particularly given the small sample size. The permutation test was therefore used to determine whether this performance reflects a true data structure or is merely an artifact of overfitting. The CV *p*-value for Q^2^ equaled 0.01, indicating that the original model performed significantly better than random permutations. The high R^2^ intercept suggests that the calibration R^2^ alone is not a reliable indicator of overfitting, whereas the negative Q^2^ intercept and the significant CV *p*-value provide evidence against overfitting [23]. However, permutation tests only allow for an assessment of overfitting within the cross-validation procedure used here and do not demonstrate the model’s robustness when applied to independent datasets.

The permutation test was performed only on the final selected model, i.e., after the choice of indicator reaction, spectral range, processing type, and number of images. Thus, it assesses the probability of randomly obtaining an equally high accuracy for this specific model, but it does not account for the multiplicity of analytical attempts made during model selection. We acknowledge this limitation and plan to systematically implement nested cross-validation in future studies, where the entire model-selection process is repeated within the training folds. However, an attempt to explore the results of performing feature selection within the permutation cycle was undertaken, as described below.

To assess the stability of the results with respect to the train/test split and the permutation strategy, we used two datasets: 6 hydronephrosis and 8 control samples (HN vs. control), and 6 VUR and 8 control samples (VUR vs. control), both analyzed with reaction A. Each dataset was divided into training (*n* = 10) and test (*n* = 4) subsets, and the procedure was repeated three times, yielding three independent random splits. All permutation tests and CV *p*-values were obtained on the training set by cross-validation; the test set (*n* = 4) was used only to report accuracy and no statistical inference was drawn from it. We varied two parameters: (1) the number of features (23 being the initial number, or 9 after selection) and (2) the permutation strategy (feature selection once vs. re-selected within each permutation) (Table 7).

**Figure 6:**
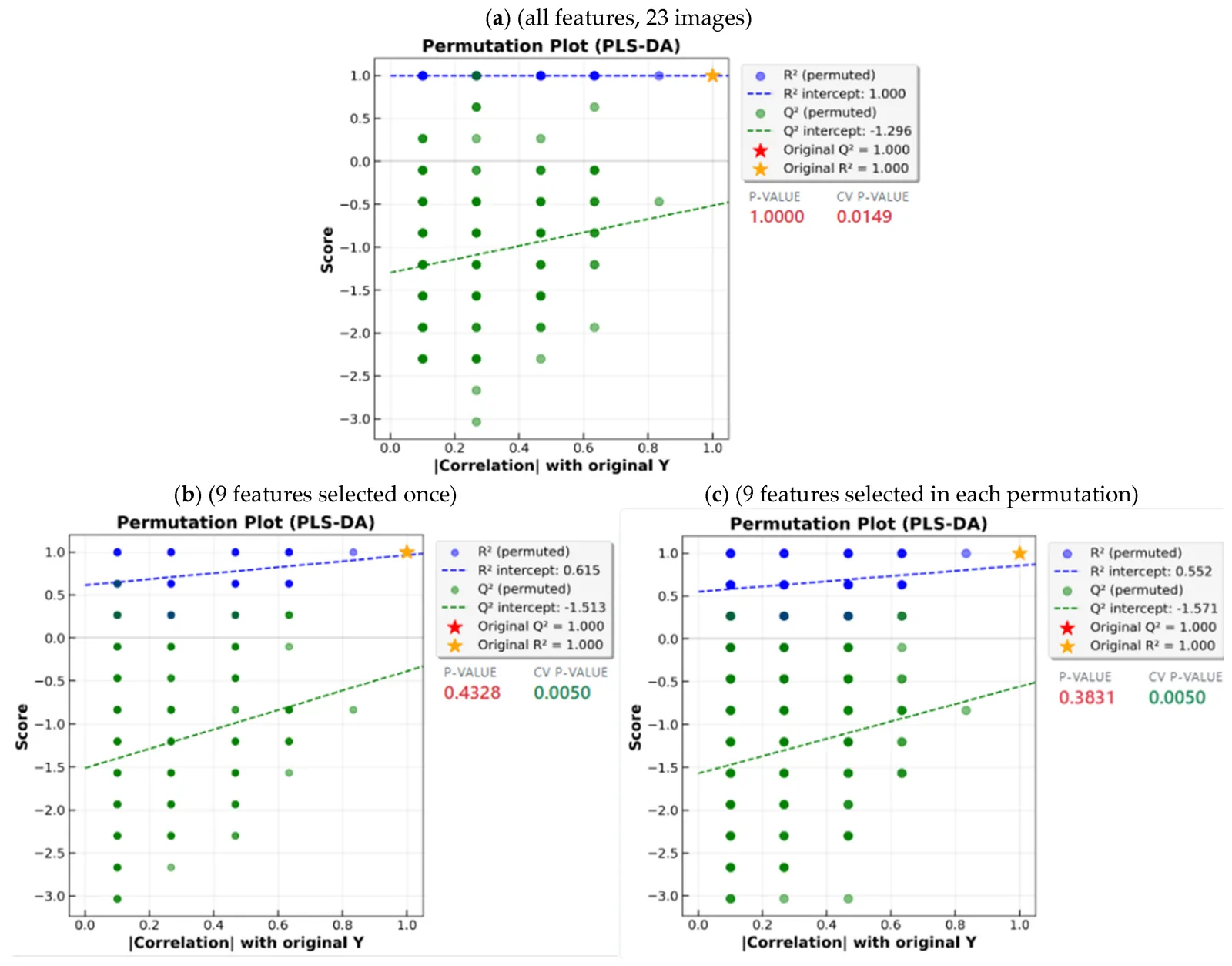
Effect of feature number: 23 features (**a**) or 9 features (**b**,**c**) and feature selection strategy: feature selection once (**b**) vs. re-selected within each permutation (**c**) on permutation-based significance (VUR vs. Control) by reaction A with PLS-DA models for dataset 3. All plots correspond to the training set composed of 5 VUR and 6 control samples. For all models, accuracy was 100% on both the training and test sets. The red star is located exactly behind the yellow star because their values are identical for both subfigures (**a**–**c**).

For HN vs. control (LDA), 23 features yielded high accuracy (mean 93% training/100% test) and significant CV *p*-values in two of three splits (0.005 and 0.02), while the third split was non-significant (*p* = 0.36). Reducing the feature set to 9 also led to a loss of significance in one split and a drop in accuracy, whereas the other two splits retained high accuracy and significance (*p* = 0.01–0.03). For the discrimination of VUR and controls using PLS-DA, both 23 and 9 features gave consistent results across all three splits with a mean accuracy of 91% (training)/83% (test) and CV *p*-values not exceeding 0.05. Thus, for VUR vs. control, the model remained statistically significant across all splits regardless of feature number or selection strategy. Notably, in both comparisons the CV *p*-values were comparable regardless of whether feature selection was performed once or re-run within each permutation, indicating that the selection strategy had minimal impact on the permutation results. The permutation plots illustrating Table 7 data for the third dataset are presented in Figure 6, while results for other datasets are given in Figures S3–S7 (Supplementary Materials), which shows the permutation plots for VUR vs. control and HN vs. control at both 23 and 9 features.

As it can be seen from the above, the results presented in Table 6 do not show evidence of overfitting in permutation tests performed within the cross-validation framework. It should be noted that these models achieved high accuracy, in some cases 100%, but with a limited number of samples (on the order of two dozen). Despite absence of overfitting, such models are unlikely to be highly robust and may, in principle, fail when applied to new samples. Nevertheless, this is a pilot study; in future work, the number of samples will be increased and new models will be constructed.

Thus, the recognition of urine samples using the kinetic optical fingerprinting method demonstrated high potential diagnostic efficiency. Indicator reactions were identified that allowed urine samples from conditionally healthy children and patients with hydronephrosis, as well as urine samples from control children and patients with vesicoureteral reflux, to be distinguished. The possibility of distinguishing all patients with uropathies (VUR or hydronephrosis) from conditionally healthy children, as well as distinguishing the two uropathies from each other, was demonstrated. At the same time, it should be noted that all recognition metrics obtained in this study pertain only to the limited set of samples investigated. Therefore, practical application of the method for diagnostic purposes will require further studies using larger sample sets.

### 3.3. Correlations of the Kinetic Optical Fingerprinting Method and Standard Clinical and Laboratory Parameters

For the indicator reactions that provided the most effective recognition of pathologies, correlations between the results and the standard clinical and laboratory parameters of the samples were investigated. Table 8 presents the correlation coefficients of the first discriminant analysis factors (or the values of the first latent variables of the PLS-DA method) with these parameters for different recognition tasks (*VUR*—*control*, *VUR*—*all other samples*, *hydronephrosis*—*control*, and *hydronephrosis*—*all other samples*) in those cases where complete recognition was achieved.

As shown in Table 8, no correlation was observed between the results of the fingerprinting method and the standard clinical and laboratory parameters. The laboratory parameters themselves were only weakly correlated with each other (the corresponding results are presented in Table S7). The absence of correlations with the results of the proposed method indicates that it provides fundamentally different information about the samples compared with standard laboratory tests. However, given the small sample size, these correlations should be interpreted as exploratory and hypothesis-generating rather than definitive evidence of independence.

## 4. Discussion

The obtained results confirm the limited diagnostic value of standard clinical and laboratory methods for the differential diagnosis of urological pathology in children. The similarity of parameter values between different groups is consistent with literature data indicating the low sensitivity of traditional laboratory markers at the early stages of urinary tract diseases [31,32]. In contrast, metabolomic analysis of urine makes it possible to detect more subtle biochemical changes associated with metabolic and inflammatory processes at the preclinical level [33,34]. A number of studies have demonstrated that metabolomic profiles can be used for the diagnosis and prediction of kidney diseases [35,36,37,38,39,40,41]. For example, different stages of renal cell carcinoma were differentiated using metabolomic analysis with an accuracy of 86–87% [36], while diabetic kidney disease [39], chronic kidney disease [40], and acute kidney injury in prenatal babies [35] were recognized with area under the receiver operating characteristic curve (AUC) values of 0.80–0.94. In our study, the typical AUC values were 0.9–1.0 (Table 6). Our results are at least comparable to those reported in the literature; however, they should be interpreted with caution due to the extremely small sample size, particularly in the hydronephrosis group (*n* = 5), which necessitates validation of the obtained classification models on an independent test cohort. In addition, the metabolomic composition of urine in young children may depend on the type of feeding, intake of vitamin preparations, and other factors, making standardization of biomaterial collection conditions necessary. A limitation of the kinetic optical fingerprinting method itself results from its high sensitivity: the reproducibility of the results on different days is limited, which complicates the formation of databases for further practical application [22]. Therefore, it is necessary to search for indicator systems that provide an optimal combination of sensitivity to a wide range of analytes and signal stability.

The authors also wish to emphasize that the proposed kinetic optical fingerprinting of urine is not a standalone diagnostic test, but a complementary, rapid, non-invasive screening tool. It does not correlate with routine parameters, reflecting a distinct molecular/metabolic level of the pathological process, thus supplementing existing diagnostic methods [34,42,43]. Requiring minimal sample preparation and yielding results in 0.5–1.5 h, it is faster than traditional methods. Its primary use is preliminary triage of pediatric patients with suspected uropathies, identifying those who need invasive procedures (e.g., VCUG or CT) and aiding monitoring. The limitations—small sample size, potential confounders (age, sex, infection status, collection conditions)—and the lack of longitudinal outcome data linking fingerprinting signals to renal function or imaging endpoints, are acknowledged. Future studies should target clinically meaningful endpoints (e.g., progression of obstruction, changes in GFR, progression on imaging) and employ longitudinal designs to establish predictive value.

## 5. Conclusions

Standard clinical and laboratory parameters in children with vesicoureteral reflux and hydronephrosis, when analyzed using nonparametric statistical methods, demonstrate substantial overlap between the groups and, even when chemometric methods are applied, provide a recognition accuracy of no more than 86%. This does not allow reliable differential diagnosis of these uropathies. In contrast, urine profiling using the kinetic optical fingerprinting method in this pilot cohort provided higher recognition accuracy, reaching 100% in some comparisons. However, these accuracy estimates are limited to the analyzed sample and may not be reproducible in larger or independent cohorts. This approach can be considered a promising direction for advanced non-invasive diagnostics in pediatric urology, but independent validation is required before any clinical application. With an increase in the number of analyzed samples, it may potentially facilitate differentiation of the type of pathology and the degree of urinary tract obstruction.

## Figures and Tables

**Table 1:** Standard clinical laboratory parameters of patients.

Sample	Used in Correlation with Indicator Reaction Data								Not Used in Correlation			
	Diagnosis	Age, yrs	Gender ^#^	ESR	Leuk	Creat	GFR	Urea	LU	PU	Sev	Lat
64	VUR	4.6	2	5	5.9	45.1	72	4.3	0	0	1	1
65	VUR	3.0	2	7	12.0	26.0	115	5.5	0	0	3	1
73	VUR	6.7	2	4	4.7	40.0	103	5.0	1	0	3	1
74	VUR	0.5	1	2	7.5	37.2	68	1.9	0	0	3	1
77	VUR	0.4	2	2	13.1	34.2	78	1.7	0	0	3	1
84	VUR	6.4	2	7	6.1	41.8	92	4.6 *	0	0	3	1
96	VUR	7.0	2	7	5.2	55.3	73	4.4	0	0	3	2
106	VUR	3.0	2	2	7.5	35.5	70	5.0	0	0	3	2
112	VUR	5.0	1	10	3.3	65.0	68	4.4	0	0	2	2
114	VUR	5.0	2	5	4.4	45.0	90	3.2	0	0	3	1
118	VUR	3.3	2	5	8.0	50.8	55	5.2	0	0	3–4	2
124	VUR	4.6	2	6	8.4	28.3	119	3.9	1	0	3	2
125	VUR	3.8	2	9	7.8	57.1	63	7.0	0	0	3	2
134	VUR	4.2	1	8	5.9	43.6	94	4.2	0	0	3	2
75	VUR	1.1	1	9	9.1	25.6	125	5.8	0	0	3	2
76	HN	1.3	2	14	12.4	71.1	48	5.4	1	0.51	2	2
105	HN	5.2	2	10	8.5	48.2	85	4.1	0	0	3	1
185	HN	5.0	2	9	4.4	30.0	114	7.5	0	0	−	1
212	HN	2.4	1	7 *	7.8	43.1	76	2.9	0	0	−	1
213	HN	3.7	1	2	6.9	47.0	80	6.3	0	0	−	1
K1	Contr	12	1	7 *	10.1	44.0 *	84	4.6 *	0	0	0	0
K13	Contr	12	1	7 *	6.9	44.7	103	4.6 *	0	0	0	0
K14	Contr	12	1	4	7.5	64.2	78	4.6 *	0	0	0	0
K15	Contr	5.0	1	18	7.7	44.6	95	5.3	0	0	0	0
K16	Contr	5.0	1	2	10.3	37.7	101	4.2	0	0	0	0
K25	Contr	3.0	1	2	6.7	42.0	86	6.1	0	0	0	0
K27	Contr	4.9	1	22	7.9	44.0	89	4.7	0	0.16	0	0
K60	Contr	5.0	2	35	4.2	33.0	84 *	4.9	0	0	0	0

* These values were missing; the column average was substituted in their place. ^#^ 1—male, 2—female; VUR—vesicoureteral reflux, HN—hydronephrosis, ESR—Erythrocyte Sedimentation Rate, mm/h; Leuk—leukocyte count in blood, ×10^3^ µL^−1^; GFR—Glomerular Filtration Rate, mL/min/1.73 m^2^; Creat—creatinine concentration in blood, µM; Urea—urea concentration in blood, mM; LU—leukocyturia; PU—proteinuria, g/L; Sev—severity of the disease; Lat—laterality (1—unilateral, 2—bilateral); a dash indicates lacking data.

**Table 2:** Conditions for conducting indicator reactions for recognizing uropathies by the kinetic fingerprint method.

Reaction	Dye	Other Reagents	Components Introduced into the Plate Well	Spectral Ranges * (Processing Type)	Number of Samples
A	1	H_2_O_2_	Phosphate buffer (pH 7.4), 30 μL; urine sample, 10 μL, water, 150 μL; CuSO_4_ 0.001 M, 30 μL; H_2_O_2_ 0.05 M, 50 μL; Dye 1, 0.04 g/L in ethanol, 30 μL	4 (no RGB-splitting)	20
B	2	Riboflavin, O_2_	Phosphate buffer (pH 7.4), 30 μL; urine sample, 10 μL; Dye 2, 0.05 g/L in water, 60 μL; Riboflavin 2.2 × 10^−5^ M, 90 μL and illuminate by blue LED (450 nm)	1, 2, 3 (no RGB-splitting)	14
C	2	NaOCl	Acetate buffer (pH 5.8), 30 μL; ethanol 95%, 30 μL; urine sample, 10 μL; Dye 2, 0.01 g/L, 100 μL; NaOCl 0.01 M, 50 μL	1 (no RGB splitting)	15
D	3	PDA **	0.1 M HCl, 60 µL; 0.0021 M PDA in ethanol, 15 µL; Dye 3, 0.01 g/L in water, 10 µL; urine sample, 5 µL	1, 4 (RGB-splitting)	15
E	4	H_2_O_2_	Phosphate buffer (pH 7.4), 30 μL; cetyltrimethylammonium bromide, 1 mM, 80 μL; urine sample, 10 μL; ethanol 95%, 110 μL; H_2_O_2_, 0.5 M, 20 μL; CuSO_4_, 0.001 M, 20 μL; Dye 4 in DMSO, 6 μM, 50 μL	4 (RGB-splitting)	15
F	2	H_2_O_2_, AuNCs ***	Phosphate buffer (pH 7.4), 30 μL; Dye 2, 0.1 g/L, 30 μL; AuNCs ***, 30 μL; urine sample, 10 μL; H_2_O_2_, 3 M, 30 μL	1, 4 (RGB-splitting)	15

* (1) Absorption/reflection of visible light; (2) fluorescence in the visible region excited at 254 nm; (3) the same, with excitation at 366 nm; (4) fluorescence in the near-IR region excited at 660 nm. ** o-Phthalaldehyde. *** Gold nanoclusters stabilized with adenosine monophosphate; gold concentration—0.2 mM [18].

**Table 3:** Accuracy of recognition of uropathies using standard laboratory parameters.

Classes to Recognize	No of Classes	Chemometric Method *						
		kNN	LDA	SR	PLS-DA	RF	SIMCA	SVM
HN, VUR, Control	3	54%	64%	71%	68%	68%	39%	57%
VUR from Control	2	67%	86%	76%	86%	86%	57%	71%
HN from Control	2	64%	73%	73%	73%	45%	55%	36%
VUR from [Control + HN]	2	71%	79%	79%	79%	79%	46%	67%
HN from [Control + VUR]	2	67%	58%	58%	58%	42%	42%	67%
HN from VUR	2	80%	65%	70%	65%	75%	70%	80%

* kNN—*k*-nearest neighbors, LDA—linear discriminant analysis, SR—softmax regression, RF—random forest, PLS-DA—partial least squares discriminant analysis, SIMCA—soft independent modelling of class analogy, SVM—support vector machine. Note: the color scale represents the recognition accuracy: green indicates the highest accuracy, red indicates the lowest accuracy, and yellow/orange represents intermediate values.

**Table 4:** Dependence of the recognition accuracy (%) of urine samples from patients with VUR and control samples on the number of processed photographs obtained in indicator reaction A.

Images Used in Processing	Number of Images	Chemometric Method						
		kNN	LDA	SR	PLS-DA	RF	SIMCA	SVM
All	23	60	67	67	100	53	87	33
2/3 *	16	60	73	67	100	60	87	33
1/2 **	12	60	93	67	100	60	60	33
1/3	8	53	100	60	100	60	73	40
1/4	6	53	80	60	80	60	53	53
1/6	4	60	87	60	87	60	60	53
First and last	2	67	60	60	60	67	47	20
First image	1	67	67	60	-	47	53	67
Last image	1	73	60	60	-	80	53	87

* Each third image (at equal time intervals) was excluded from processing. ** Each second image was disregarded. Note: the color scale represents the recognition accuracy: green indicates the highest accuracy, red indicates the lowest accuracy, and yellow/orange represents intermediate values.

**Table 5:** Conditions for processing photographic data of indicator reactions that allowed obtaining the highest accuracy values for the recognition of uropathies, presented in Table 6.

Classification Task	Spectral Ranges (1–4) *, RGB-Splitting (Y/N) **, Number of Processed Photographs (n)					
	Indicator Reaction					
	A	B	C	D	E	F
HN vs. control	4 *, N **, *n* = 23	1–3, N, *n* = 36	1, N, *n* = 9	1 + 4, Y, *n* = 4	4, Y, *n* = 7	1 + 4, Y, *n* = 4
HN vs. (control or VUR)	4, N, *n* = 23	1, N, *n* = 12	4, Y, *n* = 13	1 + 4, Y, *n* = 4	4, Y, *n* = 5	1 + 4, Y, *n* = 4
VUR vs. control	4, N, *n* = 23	1–4, Y, *n* = 47	1, Y, *n* = 13	1 + 4, Y, *n* = 4	4, Y, *n* = 4	1 + 4, Y, *n* = 4
VUR vs. (control or HN)	4, N, *n* = 1	1–3, N, *n* = 30	1, N, *n* = 13	1 + 4, Y, *n* = 2	4, Y, *n* = 4	1 + 4, Y, *n* = 4
(HN or VUR) vs. control	4, N, *n* = 12	4, Y, *n* = 11	1, N, *n* = 5	1 + 4, Y, *n* = 2	4, Y, *n* = 7	1 + 4, N, *n* = 4
HN vs. VUR	4, N, *n* = 16	1, Y, *n* = 5	1, N, *n* = 9	1 + 4, Y, *n* = 2	4, N, *n* = 7	1 + 4, Y, *n* = 4
HN vs. VUR vs. control	4, N, *n* = 12	1–3, N, *n* = 30	1, N, *n* = 13	1 + 4, Y, *n* = 2	4, Y, *n* = 7	1 + 4, Y, *n* = 4

* (1) Absorption/reflection of visible light; (2) fluorescence in the visible region excited at 254 nm; (3) the same, with excitation at 366 nm; (4) fluorescence in the near-IR region excited at 660 nm. ** Y—RGB splitting (each value represented a separate feature); N—no RGB splitting (average intensity was used).

**Table 6:** Highest accuracy of recognition of uropathies (%) achieved in the study (the values of the areas under the ROC curves (AUC) are given in parentheses for LDA method).

Pairs of Classes to Recognize		Indicator Reaction					
		A	B	C	D	E	F
HN	Control	100 (1.00)	100	100 (1.00)	100	70	91
HN	Control + VUR	85 (0.92)	100	100	93 (0.92)	73	73
VUR	Control	100	100	100 (1.00)	73	75	100 (1.00)
VUR	Control + HN	70	86 (0.88)	93 (0.93)	73	60	67
HN + VUR	Control	100	100 (1.00)	93	73	80 (0.93)	93 (0.93)
HN	VUR	91	100 (1.00)	89	100 (1.00)	63	78
HN, VUR, control (3-class)		100	100	80	86	48	53

Note. The recognition accuracies obtained by the LDA and PLS-DA methods are presented. For all results obtained by the LDA method, the AUC values obtained during cross-validation are given in parentheses. The accuracy values above 90% (“good”) are marked in red, and the values in the range of 80% to 89% (“satisfactory”) are marked in purple.

**Table 7:** Effect of feature number and feature selection strategy * on the classification accuracy and permutation-based significance (*p*-value) of discrimination of hydronephrosis from control (*n* = 14 samples) or VUR from control (*n* = 15 samples) by reaction A.

Dataset Division (Training/Test) **	23 Features		9 Features	
	CV *p*-Value	Accuracy ***	CV *p*-Value *	Accuracy
Discrimination of HN (6 samples) from control (8 samples) using LDA				
1	0.005	100%/100%	0.26/0.21	60%/50%
2	0.02	100%/100%	0.02/0.03	100%/100%
3	0.36	80%/100%	0.01/0.02	90%/100%
**Mean**	-	**93%/100%**	-	**83%/83%**
Discrimination of VUR (7 samples) from control (8 samples) using PLS-DA				
1	0.04	82%/50%	0.05/0.05	82%/50%
2	0.02	91%/100%	0.03/0.01	91%/100%
3	0.02	100%/100%	0.005/0.005	100%/100%
**Mean**	-	**91%/83%**	-	**91%/83%**

* Feature selected once/in each permutation. ** Each test dataset was composed from 2 control samples and 2 samples with uropathy. *** For training/test dataset. Note: bold text indicates the mean accuracy values for each classification task.

**Table 8:** Correlation coefficients of standard laboratory parameters of patients with the characteristics of the kinetic optical fingerprinting method.

Reaction	Discrimination Task	Variable	ESR	Leuk	Creat	GFR	Urea
B	VUR from controls	LD1 *	−0.241	0.036	0.123	−0.286	0.094
C	VUR from (controls + HN)	LV1	−0.158	−0.037	−0.224	0.132	0.193
C	HN from controls	LV1	0.308	0.313	0.222	0.220	−0.004
A	HN from (controls + VUR)	LV1	−0.209	0.175	−0.047	−0.128	−0.088

* LD1—first LDA factor obtained in processing data from the named indicator reaction; LV1—first latent variable of PLS-DA method; ESR—Erythrocyte Sedimentation Rate, mm/h; Leuk—leukocyte count in blood, ×10^3^ µL^−1^; GFR—Glomerular Filtration Rate, mL/min/1.73 m^2^; Creat—creatinine concentration in blood, µM; Urea—urea concentration in blood, mM.

## Data Availability

The original contributions presented in this study are included in the article/Supplementary Material. Further inquiries can be directed to the corresponding authors.

## Supplementary Materials

The following supporting information can be downloaded at: https://www.mdpi.com/article/10.3390/medsci14060675/s1, Figure S1: Principal component analysis score plot for standard laboratory parameters; Figure S2: Results of digitizing photographs obtained at different reaction time points; Figures S3–S7: Effect of feature number: 23 features or 9 features and feature selection strategy: feature selection once vs. re-selected within each permutation on permutation-based significance; Table S1: Accuracy of recognition of uropathies by processing optical kinetic data using a default parameter set; Table S2: Effect of spectral data range and processing type on the accuracy of uropathies recognition; Table S3: Recognition accuracy (%) of urine samples as a function of the number of processed photographs; Table S4: Cross-validation metrics that yielded the most efficient discrimination of samples by reactions A, B and C using LDA or PLS-DA; Table S5: Permutation plots for HN vs. control discrimination using reaction C by LDA: comparison of all features, manual and automatic feature selection; Table S6: Permutation plots for the chemometric models that yielded the highest accuracy in recognizing uropathies; Table S7: Correlation coefficients of standard laboratory parameters of patients with the characteristics of the method and among themselves; Table S8: Permutation plots for the chemometric models that yielded 100% accuracy in discrimination of VUR from controls by PLS-DA method using reaction A and different total number of manually chosen images processed.

## Abbreviations

The following abbreviations are used in this manuscript:

VUR	Vesicoureteral reflux
HN	Hydronephrosis
CKD	Chronic kidney disease
GFR	Glomerular filtration rate
CAKUT	Congenital Anomalies of the Kidney and Urinary Tract
kNN	*k*-nearest neighbors
LDA	Linear discriminant analysis
SR	Softmax regression
RF	Random forest
PLS-DA	Partial least squares discriminant analysis
SIMCA	Soft independent modelling of class analogy
SVM	Support vector machine
