# Supplementary material for: Fluorescence Profiling of Urine in Non-Invasive Diagnostics of Obstructive and Reflux Uropathies in Children
Source: Med Sci (Basel). 2026 Oct 15;14(6):675. doi: 10.3390/medsci14060675 (PMC13707345; doi:10.3390/medsci14060675)
Supplement: Supplementary file 1 [file medsci-14-00675-s001.zip › medsci-4545449-supplementary.pdf]

Supplementary Information to paper

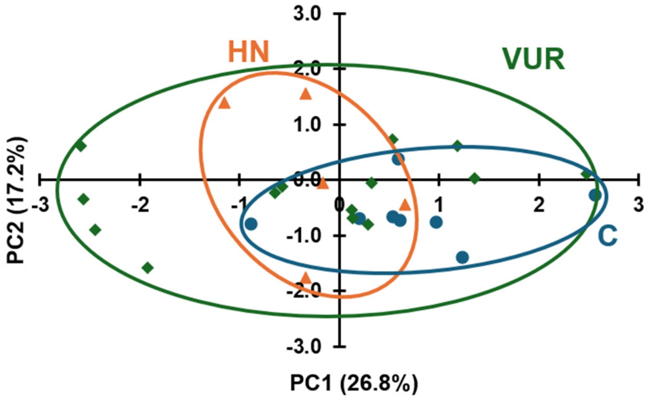

Figure S1. Principal component analysis score plot for standard laboratory parameters for recognizing uropathies (HN – hydronephrosis, VUR - vesicoureteral reflux, C – controls).

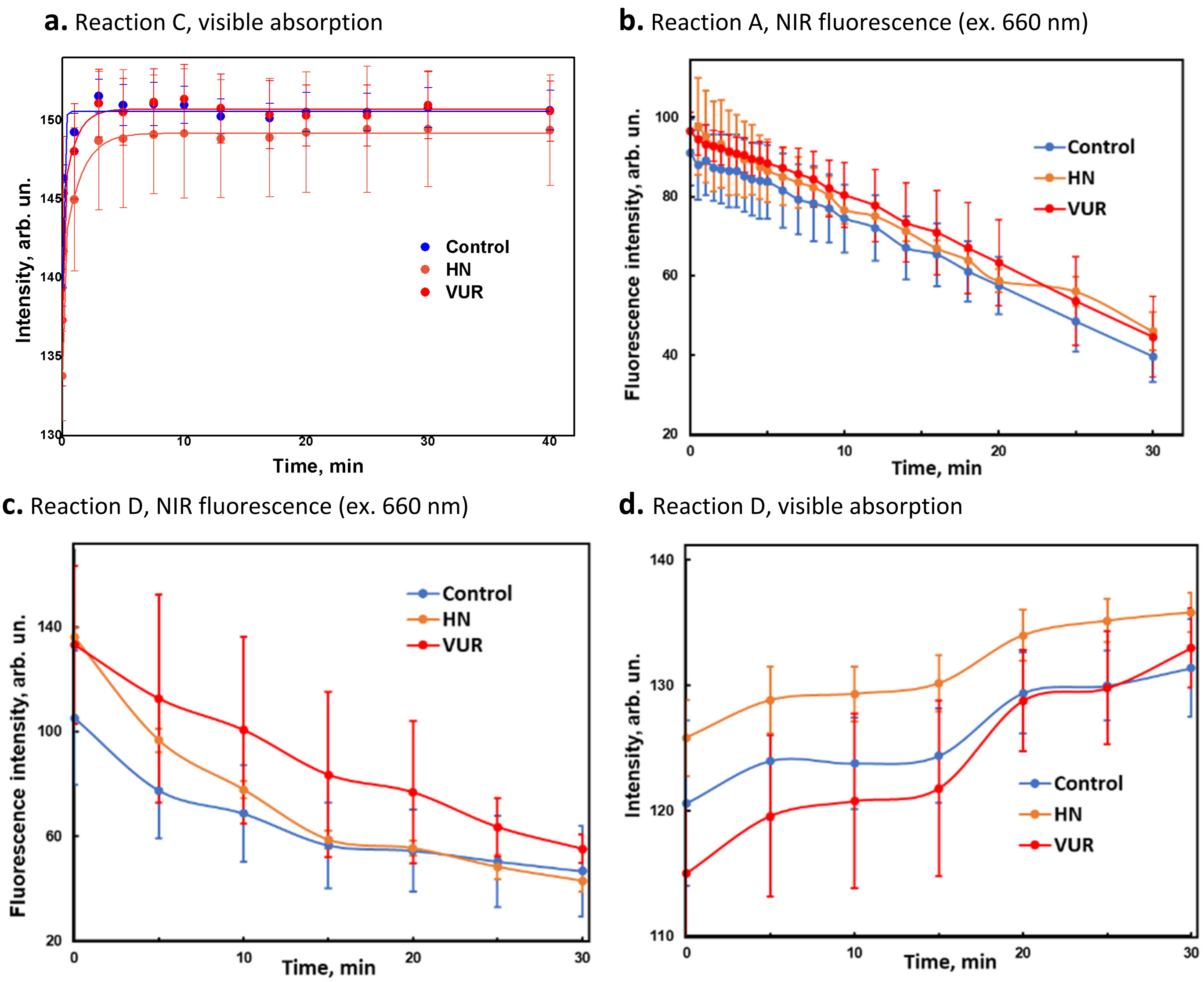

Figure S2. Results of digitizing photographs obtained at different reaction time points for reactions A, C, and D.

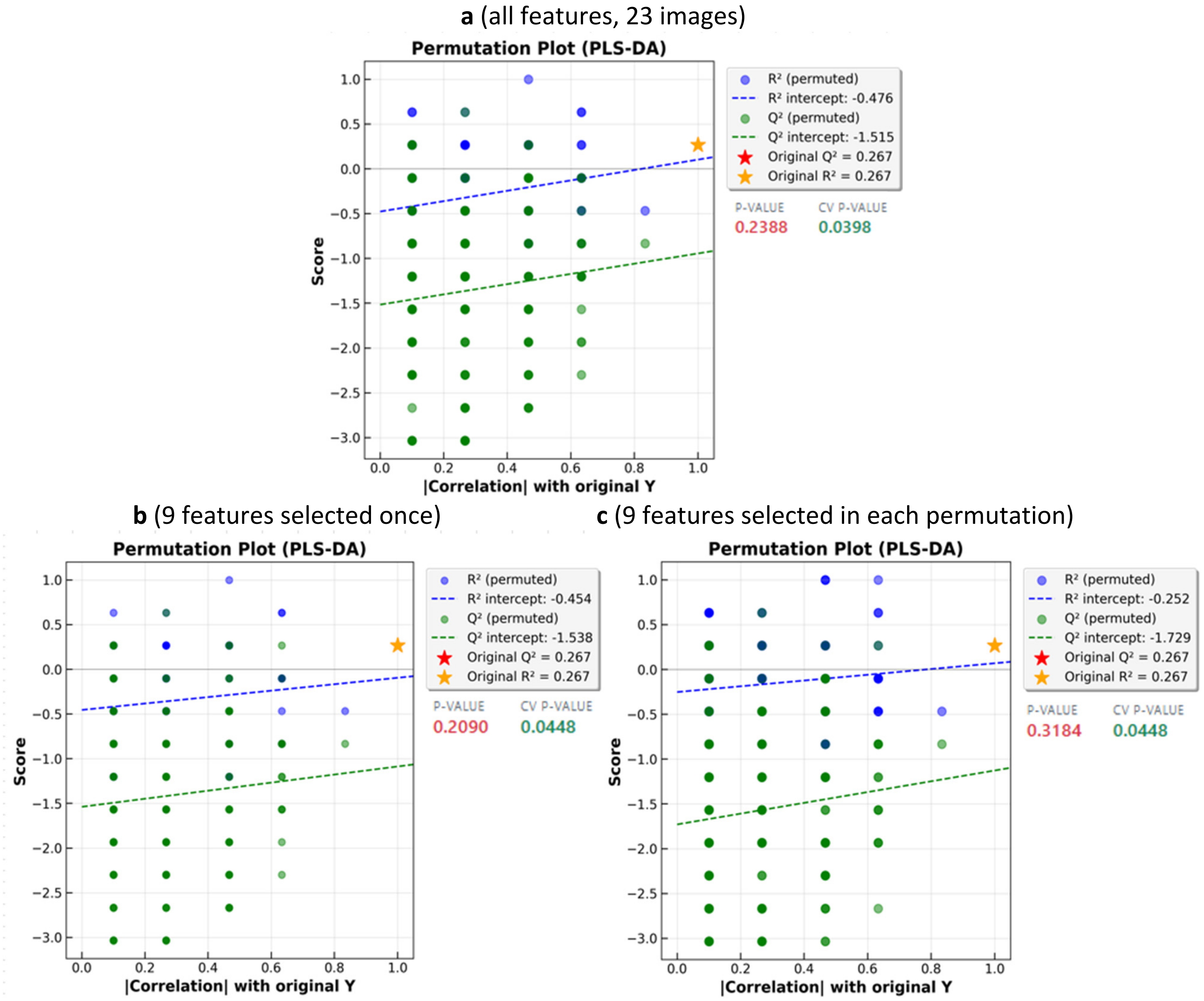

Figure S3. Effect of feature number (reaction A with PLS-DA models for dataset 1): 23 features (a) or 9 features (b, c) and feature selection strategy: feature selection once (b) vs. re-selected within each permutation (c) on permutation-based significance (VUR vs. control) by. All plots correspond to the training set composed of 5 VUR and 6 control samples. For all models, accuracy was 81% (4/5 VUR, 5/6 control) on the training set and 50% (0/2 VUR, 2/2 control) on the test set.

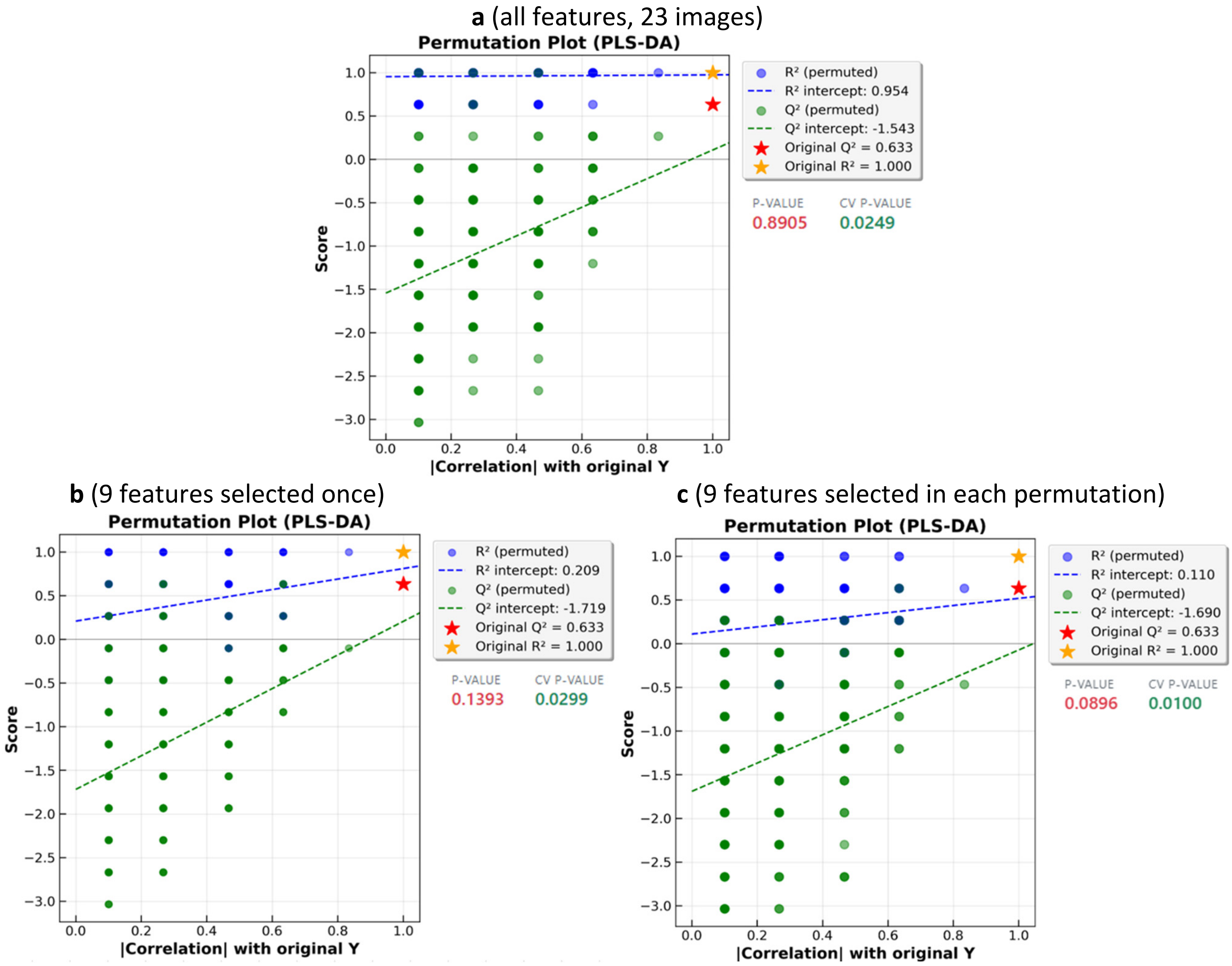

Figure S4. Effect of feature number (reaction A with PLS-DA models for dataset 2): 23 features (a) or 9 features (b, c) and feature selection strategy: feature selection once (b) vs. re-selected within each permutation (c) on permutation-based significance (VUR vs. control). All plots correspond to the training set composed of 5 VUR and 6 control samples. For all models, accuracy was 91% (4/5 VUR, 6/6 control) on the training set and 100% on the test set.

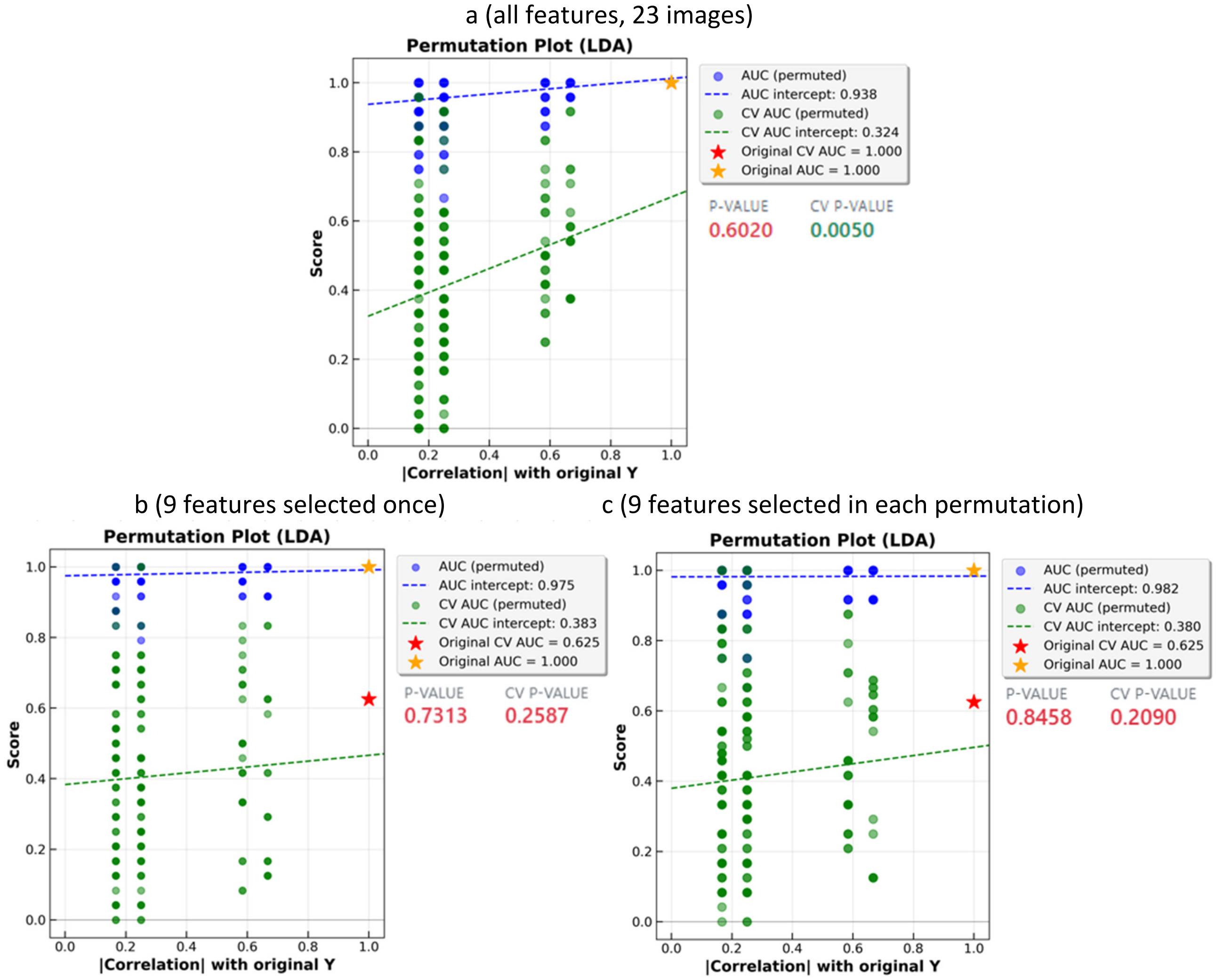

Figure S5. Effect of feature number (reaction A with LDA models for dataset 1): 23 features (a) or 9 features (b, c) and feature selection strategy: feature selection once (b) vs. re-selected within each permutation (c) on permutation-based significance (HN vs. control). All plots correspond to the training set composed of 4HN and 6 control samples. For the model using 23 features, accuracy was 100% on both the training and test sets; for the model using 9 features, accuracy was 60% on the train set (2/4 HN, 4/6 control) and 50% (1/2 HN, 1/2 control) on the test set.

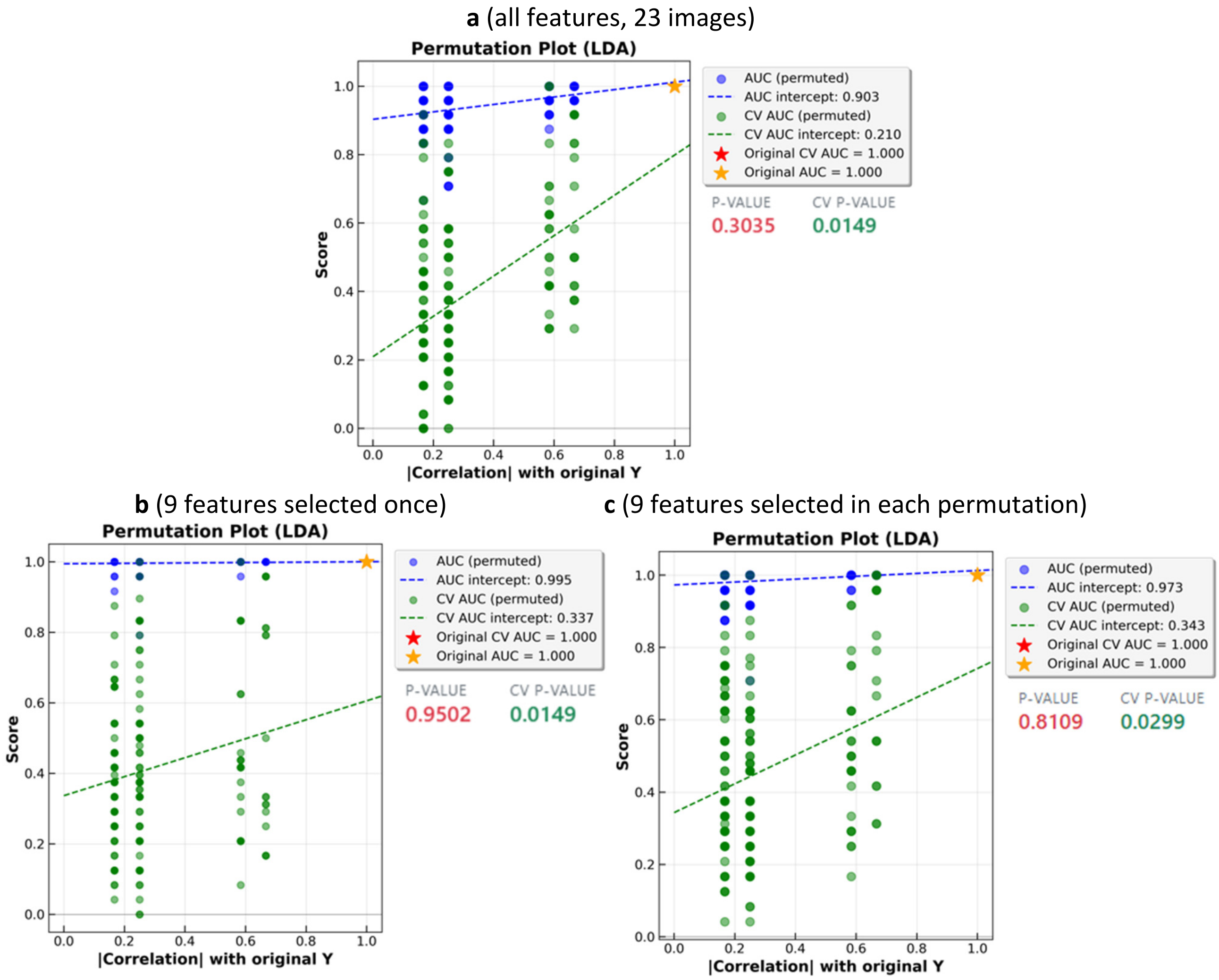

Figure S6. Effect of feature number (reaction A with LDA models for dataset 2): 23 features (a) or 9 features (b, c) and feature selection strategy: feature selection once (b) vs. re-selected within each permutation (c) on permutation-based significance (HN vs. Control). All plots correspond to the training set composed of 4 HN and 6 control samples. For all models, accuracy was 100% on both the training and test sets.

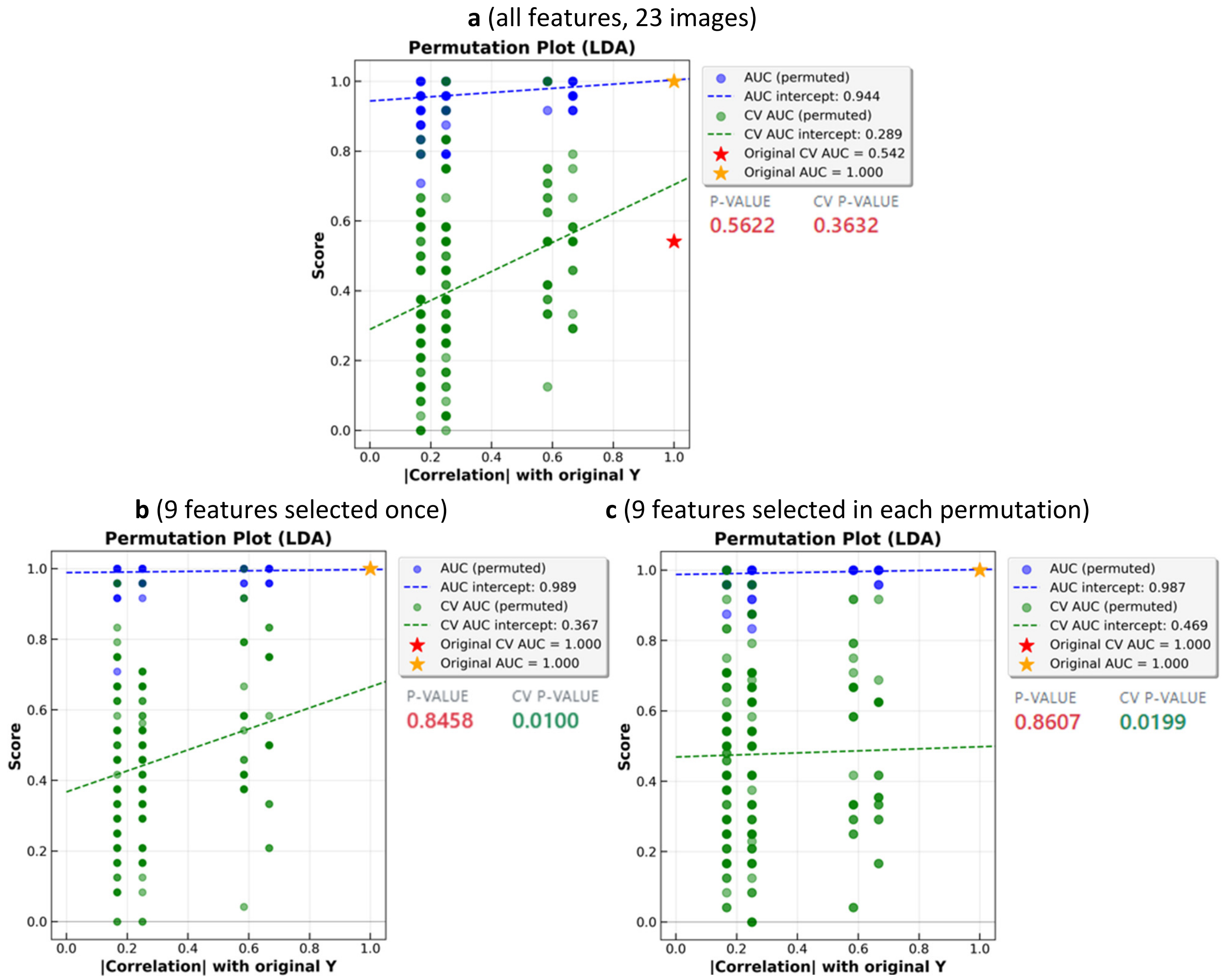

Figure S7. Effect of feature number (reaction A with LDA models for dataset 3): 23 features (a) or 9 features (b, c) and feature selection strategy: feature selection once (b) vs. re-selected within each permutation (c) on permutation-based significance (VUR vs. Control). All plots correspond to the training set composed of 4 HN and 6 control samples. For model using 23 features accuracy was 80% (2/4 HN, 6/6 control) on the training and 100% on the test set, for model using 9 features accuracy was 90% on the train set (3/4 HN, 6/6 control) and 100% on the test set.

Table S1. Accuracy of recognition of uropathies by processing optical kinetic data using a default parameter set in Chrometrica freeware

Pairs of classes for recognition		Indicator reaction					
		A	B	C	D	E	F
HN	Control	98%	99%	82%	88%	80%	88%
VUR	Control	84%	73%	74%	62%	57%	82%
HN + VUR	Control	85%	84%	80%	60%	44%	89%

Table S2. Effect of spectral data range and processing type (RGB splitting or total intensity) on the accuracy (%) of uropathies recognition using reaction C

Classification	Data range	RGB splitting	Chemometric method						
			kNN	LDA	SR	PLS-DA	RF	SIMCA	SVM
VUR / control	vis	No	40	70	70	90	50	10	50
	vis	Y	30	**100**	80	80	50	0	60
	IR	No	20	80	30	80	60	10	60
	IR	Y	20	70	70	90	50	10	60
	IR+vis	No	40	30	60	70	30	20	60
	IR+vis	Y	30	70	60	90	40	20	60
HN / other (VUR and control)	vis	No	60	47	80	80	73	67	60
	vis	Y	67	80	73	80	80	67	67
	IR	No	60	60	53	80	53	33	67
	IR	Y	60	93	60	**100**	53	33	67
	IR+vis	No	60	80	73	87	60	53	67
	IR+vis	Y	60	93	73	**100**	67	40	67

Note. The maximum accuracy values obtained by models that passed the permutation test for non-overfitting are boldfaced.

kNN – k-nearest neighbors, LDA – linear discriminant analysis, SR – softmax regression, RF – random forest, PLS-DA – partial least squares discriminant analysis, SIMCA – soft independent modelling of class analogy, SVM – support vector machine.

Table S3. Recognition accuracy (%) of urine samples from patients with vesicoureteral reflux or hydronephrosis and control samples as a function of the number of processed photographs captured during reaction C (visible range and RGB-splitting were selected for processing)

Images used in processing	Total number of images processed	Chemometric method						
		kNN	LDA	SR	PLS-DA	RF	SIMCA	SVM
All	13	60	87	87	87	67	80	60
2/3*	9	60	87	87	87	80	67	60
1/2**	7	60	67	87	87	80	80	60
1/3	5	60	67	87	93	73	73	60
1/4	4	60	73	87	87	73	73	60
1/6	3	60	80	87	87	87	80	60
First and last	2	73	87	87	87	80	80	60
First image	1	87	87	87	87	80	60	47
Last image	1	53	40	40	53	53	60	60

* Each third image (at equal time intervals) was excluded from processing.

** Each second image was disregarded.

Table S4. Accuracy, balanced accuracy, AUC, sensitivity, and specificity with 95% confidence intervals (calculated using the Clopper–Pearson exact method for accuracy, the score-type interval of Newcombe for AUC) for the classification tasks that yielded the most efficient discrimination of samples by reactions A, B and C using LDA or PLS-DA; for each metric and task, the first row shows the point estimate; for sensitivity and specificity the fraction of correctly classified cases (correct / total) is shown next to it; the second row shows the 95% confidence interval

Reaction A					
**CV metric**	**HN vs. control, LDA**	**VUR vs. control, PLS-DA**	**(HN or VUR) vs. control, PLS-DA**	**HN vs. VUR, PLS-DA**	**HN vs. VUR vs. control, PLS-DA**
Accuracy	1.00<br>0.77–1.00	1.00<br>0.78–1.00	1.00<br>0.83–1.00	1.91<br>0.59–1.00	1.00<br>0.82–1.00
Balanced Accuracy	1.00<br>0.71–1.00	1.00<br>0.72–1.00	1.00<br>0.77–1.00	0.90<br>0.55–1.00	1.00<br>0.74–1.00
AUC	1.00<br>0.72–1.00	1.00<br>0.74–1.00	1.00<br>0.79–1.00	0.87<br>0.51–0.97	-*
Sensitivity	1.00 6/6<br>0.54–1.00	1.00 7/7<br>0.59–1.00	1.00 12/12<br>0.73–1.00	.00 6/6<br>0.54–1.00	-
Specificity	1.00 8/8<br>0.63–1.00	1.00 8/8<br>0.63–1.00	1.00 8/8<br>0.63–1.00	0.80 4/5<br>0.28–1.00	-
Reaction B					
**CV metric**	**HN vs. (VUR or control), PLS-DA**	**VUR vs. control, PLS-DA**	**(HN or VUR) vs. control, LDA**	**HN vs. VUR, LDA**	**HN vs. VUR vs. control, PLS-DA**
Accuracy	1.00<br>0.77–1.00	1.00<br>0.74–1.00	1.00<br>0.77–1.00	1.00<br>0.59–1.00	1.00<br>0.75–1.00
Balanced Accuracy	1.00<br>0.62–1.00	1.00<br>0.67–1.00	1.00<br>0.71–1.00	1.00<br>0.50–1.00	1.00<br>0.64–1.00
AUC	1.00<br>0.63–1.00	1.00<br>0.69–1.00	1.00<br>0.72–1.00	1.00<br>0.49–1.00	-
Sensitivity	1.00 3/3<br>0.29–1.00	1.00 6/6<br>0.54–1.00	1.00 8/8<br>0.63–1.00	1.00 5/5<br>0.48–1.00	-
Specificity	1.00 11/11<br>0.72–1.00	1.00 6/6<br>0.54–1.00	1.00 6/6<br>0.54–1.00	1.00 2/2<br>0.16–1.00	-
Reaction C					
**CV metric**	**HN vs. control, LDA**	**HN vs. (VUR or control), PLS-DA**	**VUR vs. control, LDA**	**VUR vs. (HN or control), LDA**	**(HN or VUR) vs. control, PLS-DA**
Accuracy	1.00<br>0.72–1.00	1.00<br>0.78–1.00	1.00<br>0.69–1.00	0.93<br>0.68–1.00	0.93<br>0.68–1.00
Balanced Accuracy	1.00<br>0.65–1.00	1.00<br>0.70–1.00	1.00<br>0.62–1.00	0.95<br>0.61–1.00	0.94<br>0.65–1.00
AUC	1.00<br>0.67–1.00	1.00<br>0.72–1.00	1.00<br>0.63–1.00	0.93<br>0.60–1.00	1.00<br>0.73–1.00
Sensitivity	1.00 5/5<br>0.48–1.00	1.00 5/5<br>0.48–1.00	1.00 4/4<br>0.40–1.00	1.00 4/4<br>0.40–1.00	0.89 8/9<br>0.52–1.00
Specificity	1.00 6/6<br>0.54–1.00	1.00 10/10<br>0.69–1.00	1.00 6/6<br>0.54–1.00	0.91 10/11<br>0.59–1.00	1.00 6/6<br>0.54–1.00

* The metric was not calculated for the task.

Table S5. Permutation plots for HN vs. control discrimination using reaction C and LDA: all features – no selection (a), manual feature selection (b), automatic feature selection (c, d)

(a) 13 features (all features), 91%

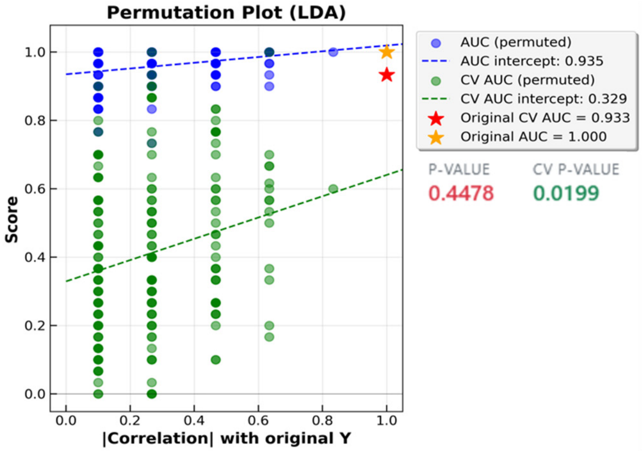

(b) 9 features (manual), 100%

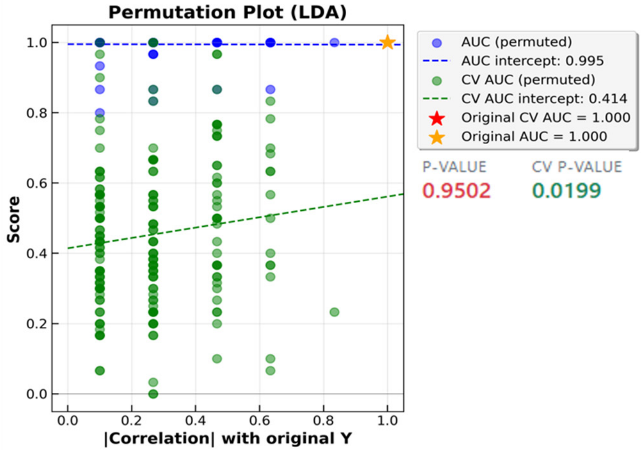

(c) 9 features (auto), 100%

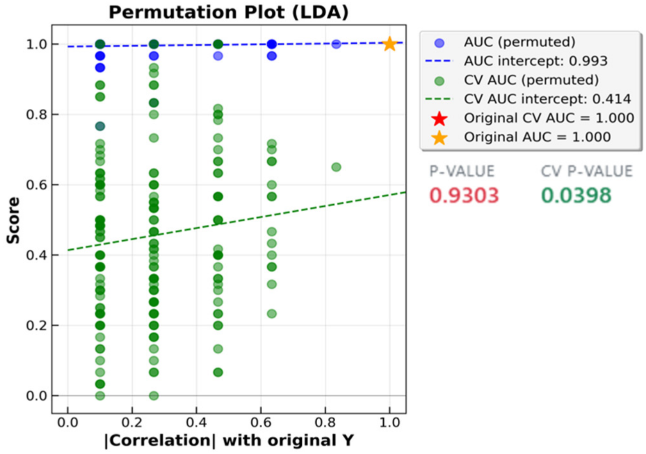

(d) 3 features (auto), 100%

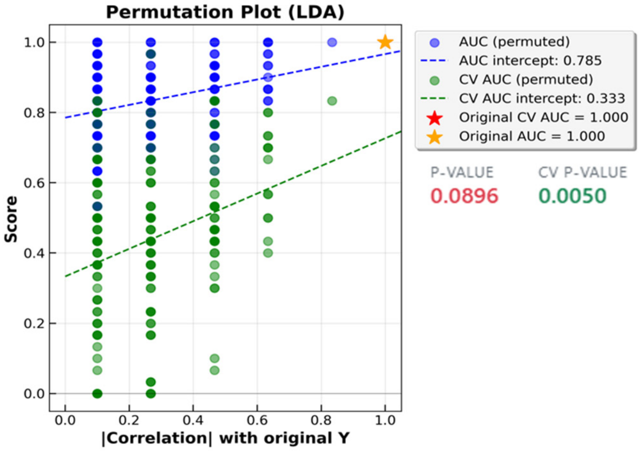

Note. Manual selection: every third visible-range photograph was removed, leaving 9 images. Automatic selection was made using Chrometrica software, which ranked features by the ratio of between-class to within-class variance and retained the highest-ranked ones (9 or 3 features out of 13). Automatic feature selection was performed anew in each permutation, whereas manual selection was applied once to the original data. Features were selected for each permutation. Classification task, accuracy (%) and total number of images processed are indicated above the plots. Accuracy is shown above each panel; *p*-values and cross-validated *p*-values are given below the plot legends. Cross-validated *p*-value was estimated as the number of permutations with scores exceeding original Q^2 or CV AUC value to the total number of permutations (200).

Table S6. Permutation plots for the chemometric models that yielded the highest accuracy in recognizing uropathies using reaction A. Classification task, accuracy (%) and total number of images processed are indicated above the plots. The original values of Q^2 (for PLS-DA) or CV AUC (for LDA) for unpermuted data are shown in the plot legend; *p*-value and cross-validation *p*-value are shown below the plot legend

HN / control: 100%, 23 images

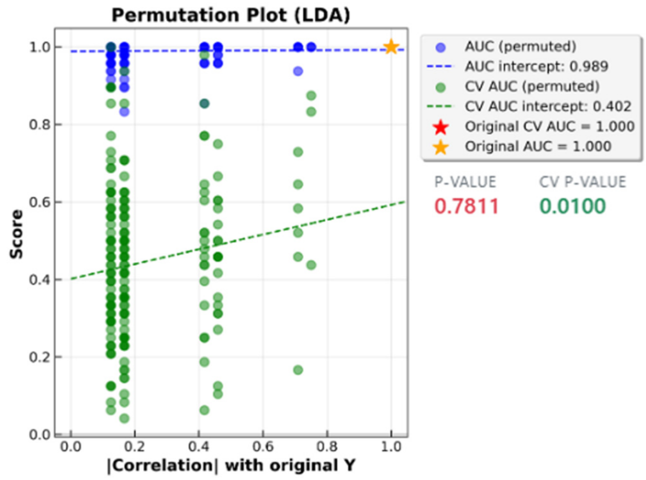

HN / (control + VUR): 85%, 23 images

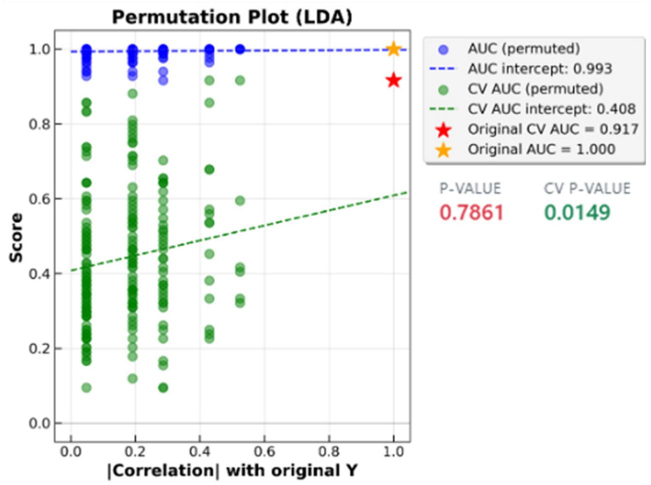

VUR / control: 100%, 23 images

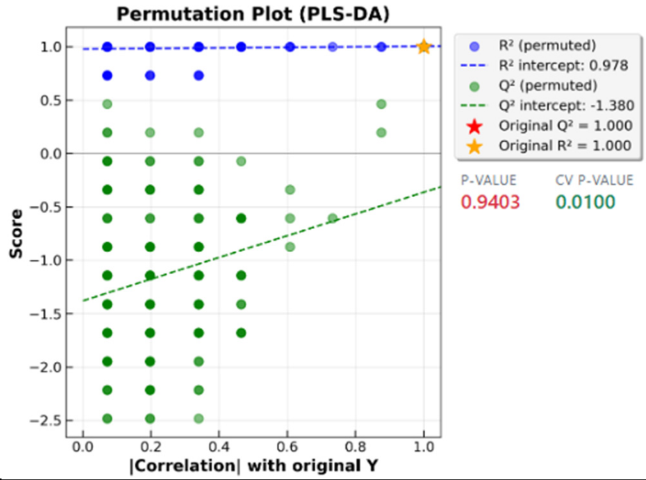

VUR / (control + HN): The maximum accuracy value did not exceed 80%.

(VUR + HN) / control: 100%, 12 images

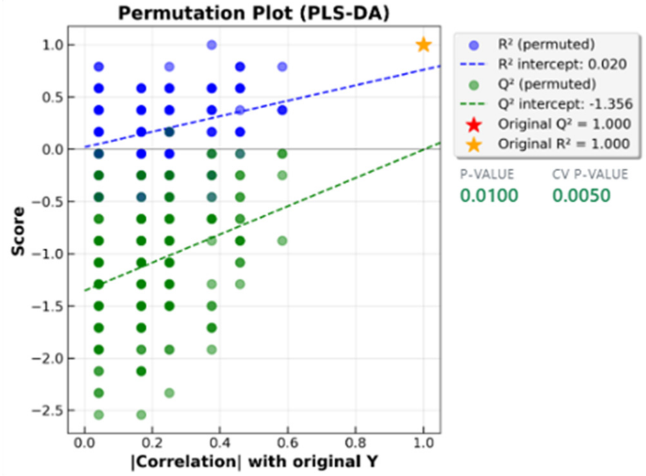

VUR / HN: 91%, 16 images

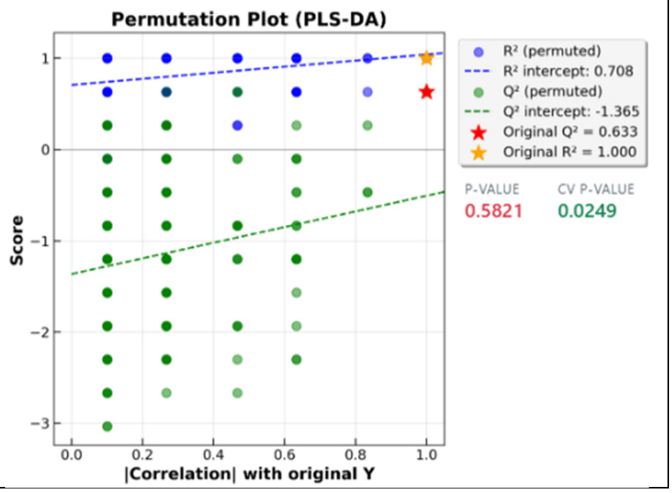

Note. Permutation plots for other reactions are similar and available from authors as a MS Excel file on request.

Table S7. Correlation coefficients of standard laboratory parameters of patients with the characteristics of the fingerprinting method and among themselves

Variable	LD1 or LV1*	ESR	Leukoc	Creat	GFR	Urea
Reaction B, discrimination of VUR from controls						
LD1	1	-0.241	0.036	0.123	-0.286	0.094
ESR	-0.241	1	0.279	0.061	-0.010	0.014
Leukoc	0.036	0.279	1	0.331	-0.370	0.146
Creat	0.123	0.061	0.331	1	**-0.836**	0.351
GFR	-0.286	-0.010	-0.370	**-0.836**	1	-0.524
Urea	0.094	0.014	0.146	0.351	-0.524	1
Reaction C, discrimination of VUR from (controls + HN)						
LV1	1	-0.158	-0.037	-0.224	0.132	0.193
ESR	-0.158	1	-0.447	-0.191	0.042	0.178
Leukoc	-0.037	-0.447	1	-0.167	0.144	-0.273
Creat	-0.224	-0.191	-0.167	1	**-0.697**	0.066
GFR	0.132	0.042	0.144	**-0.697**	1	0.197
Urea	0.193	0.178	-0.273	0.066	0.197	1
Reaction A, discrimination of HN from (controls + VUR)						
LV1		-0.209	0.175	-0.047	-0.128	-0.088
ESR	-0.209		-0.210	-0.078	0.021	0.108
Leukoc	0.175	-0.210		0.181	-0.197	-0.159
Creat	-0.047	-0.078	0.181		**-0.753**	0.102
GFR	-0.128	0.021	-0.197	**-0.753**		0.075
Urea	-0.088	0.108	-0.159	0.102	0.075	
Reaction C, discrimination of HN from controls						
LV1		0.308	0.313	0.222	0.220	-0.004
ESR	0.308		-0.553	-0.332	0.124	0.187
Leukoc	0.313	-0.553		0.354	-0.223	-0.455
Creat	0.222	-0.332	0.354		**-0.405**	-0.134
GFR	0.220	0.124	-0.223	**-0.405**		0.671
Urea	-0.004	0.187	-0.455	-0.134	0.671	

* LD1 – first LDA factor obtained in processing data from the named indicator reaction; LV1 – first latent variable of PLS-DA method; ESR – Erythrocyte Sedimentation Rate, mm/h; Leukoc – leukocyte count in blood, × 10^3 μL^{-1}; GFR – Glomerular Filtration Rate, mL/min/1.73 m²; Creat – creatinine concentration in blood, µM; Urea – urea concentration in blood, mM.

Note. Differences in the correlation coefficients between standard parameters are due to the unequal number of samples, which, in turn, is caused by different recognition tasks (whether one of the pathologies differs from all other samples or only from the controls).

Table S8. Permutation plots for the chemometric models that yielded 100% accuracy in discrimination of VUR from controls by PLS-DA method using reaction A and different total number of manually chosen images processed. Total number of images processed is indicated above the plots. The original values of Q^2 for unpermuted data are shown in the plot legend; p-value and cross-validation p-value are shown below the plot legend

23 images

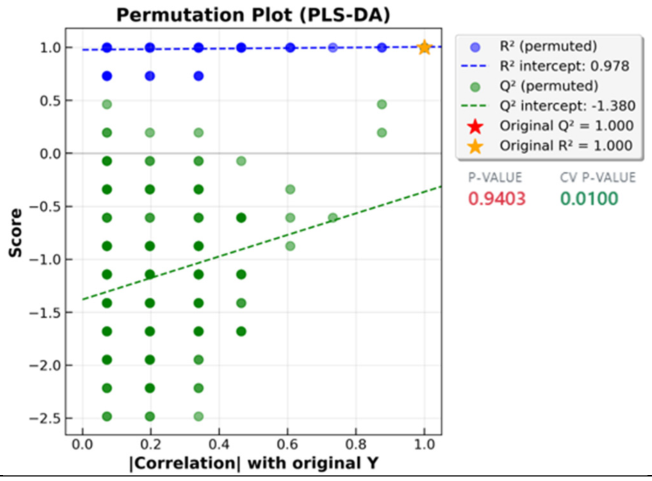

16 images

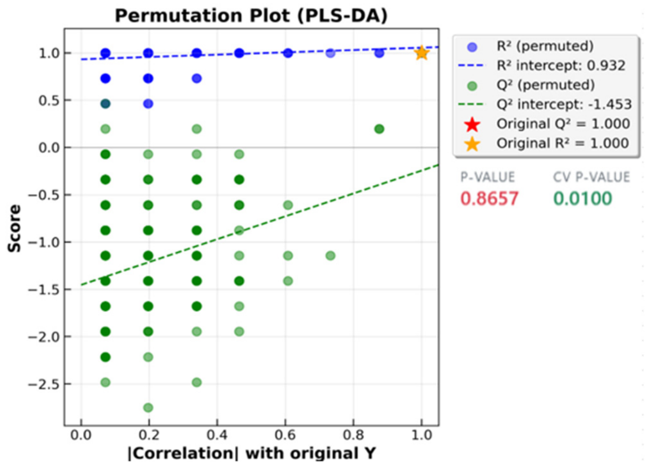

12 images

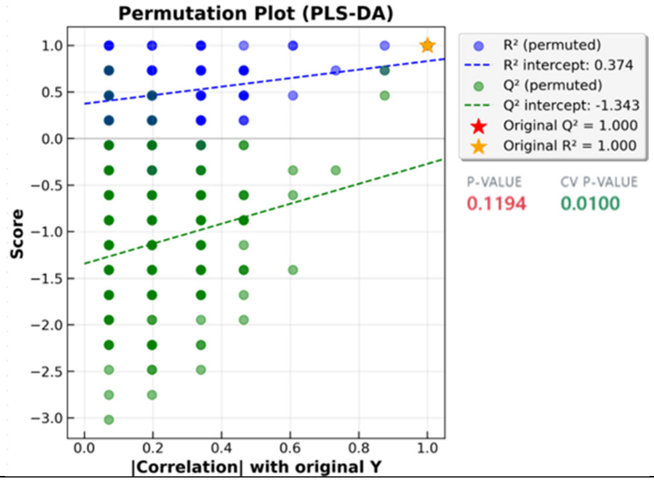

8 images

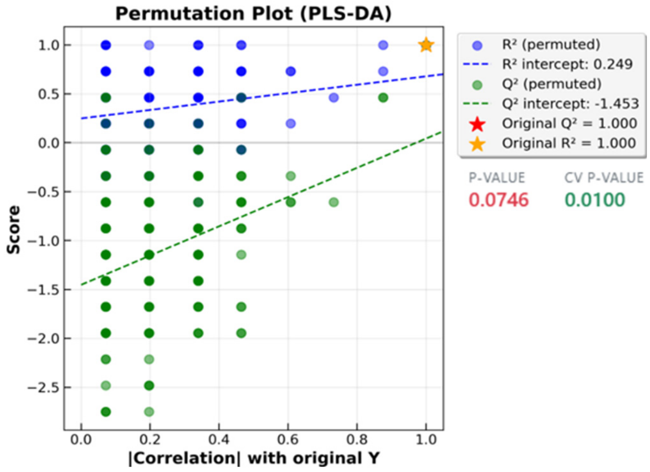

* Cross-validated *p*-value was estimated as the number of permutations with scores exceeding original Q^2 or CV AUC value to the total number of permutations (200).
